# Astrocytes as Key Regulators of Brain Energy Metabolism: New Therapeutic Perspectives

**DOI:** 10.3389/fphys.2021.825816

**Published:** 2022-01-11

**Authors:** Elidie Beard, Sylvain Lengacher, Sara Dias, Pierre J. Magistretti, Charles Finsterwald

**Affiliations:** GliaPharm SA, Geneva, Switzerland

**Keywords:** astrocytes, lactate, glucose, brain, energy, metabolism, new therapeutic approach, GliaPharm

## Abstract

Astrocytes play key roles in the regulation of brain energy metabolism, which has a major impact on brain functions, including memory, neuroprotection, resistance to oxidative stress and homeostatic tone. Energy demands of the brain are very large, as they continuously account for 20–25% of the whole body’s energy consumption. Energy supply of the brain is tightly linked to neuronal activity, providing the origin of the signals detected by the widely used functional brain imaging techniques such as functional magnetic resonance imaging and positron emission tomography. In particular, neuroenergetic coupling is regulated by astrocytes through glutamate uptake that triggers astrocytic aerobic glycolysis and leads to glucose uptake and lactate release, a mechanism known as the Astrocyte Neuron Lactate Shuttle. Other neurotransmitters such as noradrenaline and Vasoactive Intestinal Peptide mobilize glycogen, the reserve for glucose exclusively localized in astrocytes, also resulting in lactate release. Lactate is then transferred to neurons where it is used, after conversion to pyruvate, as a rapid energy substrate, and also as a signal that modulates neuronal excitability, homeostasis, and the expression of survival and plasticity genes. Importantly, glycolysis in astrocytes and more generally cerebral glucose metabolism progressively deteriorate in aging and age-associated neurodegenerative diseases such as Alzheimer’s disease. This decreased glycolysis actually represents a common feature of several neurological pathologies. Here, we review the critical role of astrocytes in the regulation of brain energy metabolism, and how dysregulation of astrocyte-mediated metabolic pathways is involved in brain hypometabolism. Further, we summarize recent efforts at preclinical and clinical stages to target brain hypometabolism for the development of new therapeutic interventions in age-related neurodegenerative diseases.

## Introduction

The brain requires high amounts of energy to function. As a result, 20–25% of the energy consumed by the human body is dedicated to cerebral functions, although the brain only represents 2% of the total body mass. Maintenance and restoration of neuronal ion gradients and synaptic transmission, as well as uptake and recycling of neurotransmitters are the major contributors to these energy demands (Riveros et al., 1986; Wong-Riley, 1989; Attwell and Laughlin, 2001; Alle et al., 2009; Hyder et al., 2013; Magistretti and Allaman, 2016; Yu et al., 2018). Glucose is the main energy substrate in the adult brain. However, other sources of energy can be used under particular circumstances, such as ketone bodies that are consumed during development and fasting, and lactate that can be preferentially used during periods of intense physical activity (Owen et al., 1967; Nehlig, 2004; Rasmussen et al., 2011; Chowdhury et al., 2014; Magistretti and Allaman, 2018). Importantly, when plasma lactate concentrations rise, central nervous system (CNS) lactate levels also increase, which is correlated with decreased glucose uptake, and indicating a preferential utilization of lactate over glucose as brain energy source (Smith et al., 2003).

Several neurodegenerative diseases linked to aging are characterized by a decrease in the consumption of energy by the brain in specific regions. These include, among others, Alzheimer’s disease (AD), Parkinson’s disease (PD), Frontotemporal dementia (FTD), Amyotrophic lateral sclerosis (ALS), depression and certain neurodevelopmental disorders. Hypometabolism also occurs in physiological aging, a fact that may participate in the vulnerability of the nervous system to pathological states of aging. Decreased energy availability for neurons results in neurodegeneration, cognitive impairment, as well as abnormalities in neuronal function and excitability (Muddapu et al., 2020). Astrocytes, a type of glial cells in the brain, support essential functions such as maintenance of neurotransmitter pools, trophic support, metabolism, synaptic formation and plasticity, myelin sheath formation, injury healing, and immune surveillance (Burda et al., 2016; Manninen et al., 2020). They are key in regulating neurometabolic and neurovascular couplings, thereby linking neuronal activity to brain energy consumption. In particular, astrocytes respond to neuronal activity by taking up glutamate at the synapse, which triggers aerobic glycolysis and lactate secretion. Then, lactate can be used by neurons as preferred energy source upon activity, as formulated by the astrocyte-neuron lactate shuttle (ANLS) model (Pellerin and Magistretti, 1994; Belanger et al., 2011; Magistretti and Allaman, 2018). Astrocytes also modulate activity-dependent vasodilation through nitric oxide-mediated pathways (Bonvento et al., 2002).

In this review, we discuss the different roles played by astrocytes in the control of brain energy metabolism and homeostasis, and how these pathways are affected in aging and hypometabolic neurodegenerative diseases such as AD. Further, we review the current therapeutic strategies from *in vitro*, *in vivo*, and clinical evidence that aim at restoring brain energy deficits in neuropathologies with metabolic dysfunctions.

## Astrocyte-Mediated Metabolic Support

Under normal homeostatic conditions, the supply and demand of energy are tightly coupled. For instance, cerebral blood flow (CBF) and glucose utilization increase in response to neuronal activity through processes known as neurovascular and neurometabolic couplings (Belanger et al., 2011). These processes constitute the bases of functional brain imaging techniques, among which positron emission tomography (PET) that allows determination of CBF, cerebral metabolic rate of glucose consumption (CMRglc), cerebral metabolic rate of oxygen consumption CMRO2, as well as functional magnetic resonance imaging (fMRI) that measures brain oxygenation and blood volume (Magistretti and Pellerin, 1996; Raichle and Mintun, 2006; Figley and Stroman, 2011; Roumes et al., 2021).

Astrocytes have unique cytoarchitectural features that ideally position them to sense their surrounding environment and dynamically respond to extracellular changes (Belanger et al., 2011). They possess numerous processes that form highly organized anatomical domains interconnected through functional networks via gap junctions. Some of these processes closely ensheath synapses, whereas others are in contact with brain capillaries (Iadecola and Nedergaard, 2007; Oberheim et al., 2009; Mathiisen et al., 2010). At the synapse level, astrocytes’ perisynaptic processes express glutamate transporters that can sense changes in neuronal activity, while at the vasculature level, luminal surface of their endfeet that is in contact with vascular endothelium express glucose transporter 1 (GLUT1) (Patching, 2017), that will allow facilitated diffusion of glucose into astrocytes, to supply energy upon neuronal activity. Finally, astrocytes can release vasoactive substances to act on brain glucose supply depending on neuronal activity state (Belanger et al., 2011; MacVicar and Newman, 2015).

Neurons and astrocytes possess distinct metabolic profiles. In the presence of oxygen, neurons process glucose in an oxidative way to yield ATP through mitochondrial activity, while glucose entering astrocytes preferentially undergoes glycolysis to produce pyruvate and lactate (Magistretti and Allaman, 2015; Supplie et al., 2017). Astrocytes specifically express glycolytic enzymes, which make them utilize 80% of the glucose through glycolysis. In neurons, glycolytic enzymes, such as 6-phosphofructo-2-kinase/fructose-2,6-biphosphatase 3 (PFKFB3) and pyruvate dehydrogenase kinase 4 (PDK4) are inhibited, which make them highly phosphorylative cells (Magistretti and Allaman, 2018). Furthermore, astrocytes preferentially express lactate dehydrogenase 5 (LDH5), which favors the conversion of pyruvate into lactate, while neurons exclusively express lactate dehydrogenase 1 (LDH1) that favors conversion of lactate into pyruvate (Bittar et al., 1996). Astrocytes also have higher NADH to NAD^+^ ratio than neurons, which favors the reduction of pyruvate into lactate (Mongeon et al., 2016). Interestingly, inhibiting mitochondrial activity specifically in astrocytes did not have any phenotypic effect in mice (Supplie et al., 2017). In contrast, enhancing glycolysis in neurons led to dramatic decrease in glucose utilization in the pentose phosphate pathway, increased oxidative stress and apoptosis (Herrero-Mendez et al., 2009). These studies highlight the cellular specificity of distinct metabolic pathways in the brain with astrocytes being predominantly glycolytic, while neurons are oxidative.

As formulated by the ANLS, glutamate is taken up by astrocytes and recycled through the glutamate-glutamine cycle (Bak et al., 2006; McKenna, 2007). This process, which is mediated by astrocytic Na^+^-dependent glutamate transporters, leads to increases in cytosolic Na^+^ that activates Na^+^/K^+^ ATPase, thereby increasing ATP consumption (Magistretti and Chatton, 2005) and stimulation of GLUT1 activity (Porras et al., 2008). In turn, glycolysis is activated and results in enhanced glucose uptake in astrocytes and release of lactate toward neurons (Pellerin and Magistretti, 2012). Under resting conditions, astrocytes release 85% of the glucose they consume in the form of lactate (Bolanos et al., 1994). An *in vivo* study using two photon microscopy and lactate fluorescence resonance energy transfer (FRET) nanosensors confirmed lactate gradient between astrocytes and neurons (Machler et al., 2016). Another level of astrocyte-neuron metabolic coupling is through the activity-dependent production of NH4+ in neurons that, upon transfer to astrocytes, favors astrocytic glycolysis. Thus, in neurons, conversion of glutamine into glutamate by phosphate activated glutaminase (PAG) leads to the production of NH4+, which can be transferred to astrocytes through transporters and K^+^ channels (Kelly and Rose, 2010). In astrocytes, NH4+ can enter the mitochondria and acidifies mitochondrial matrix, which in turn inhibits mitochondrial incorporation of pyruvate that depends on the H^+^-coupled mitochondrial pyruvate carrier (MPC) (Herzig et al., 2012; Lerchundi et al., 2015).

Lactate is a metabolic end-product that cannot directly be used and requires its conversion into pyruvate to serve as energy and carbon source to the tricarboxylic acid (TCA) cycle (Barros et al., 2020). One of the advantages of producing lactate that is not readily consumed is to allow its distribution and exchanges between lactate producing and lactate consuming cells (Brooks, 2018). Importantly, lactate also serves as a signaling molecule that modulates mechanisms underlying synaptic plasticity and memory consolidation through the regulation of plasticity genes expression (Suzuki et al., 2011; Yang et al., 2014; Margineanu et al., 2018). Neuroprotective effects of lactate have been demonstrated in various types of brain damages, including ischemic (Schurr et al., 2001; Smith et al., 2003), excitotoxic and mechanical insults (Ros et al., 2001; Cureton et al., 2010). The transfer of lactate between cells is specific and controlled by monocarboxylate transporters (MCTs). There are different types of MCTs that are differentially expressed between producing and receiving cells and have different affinities for lactate. For instance, neurons exclusively express high-affinity MCT2, while astrocytes express lower-affinity MCT1 and MCT4 (Roosterman and Cottrell, 2020). Some studies shown that MCT2 expression in neurons is co-localized with glutamate receptors at the postsynaptic membranes of fast acting excitatory synapses, further supporting the intracellular signaling roles of lactate (Bergersen et al., 2001, 2005). Since lactate is co-transported through MCTs with H^+^, regulation of pH is essential for the transport of lactate (Bosshart et al., 2019). Lactate symbiosis between astrocytes and neurons is also well demonstrated through the role of energy sensor AMP-activated protein kinase (AMPK). Thus, intracerebral levels of lactate were found to be decreased in AMPK-deficient mice, which was concomitant with decreased glycolysis, oxidative phosphorylation and neuronal survival (Muraleedharan et al., 2020). Mechanistically, phosphorylation of AMPK in astrocytes was found to destabilize thioredoxin-interaction protein (TXNIP), which led to the translocation of GLUT1 at the plasma membrane, glucose uptake and lactate production that in turn provided neuroprotection in a non-cell-autonomous manner (Muraleedharan et al., 2020). The lactate signaling may also occur through the activation of the lactate responsive-G-protein-coupled receptor 81 (GPR81) (Lauritzen et al., 2014; Morland et al., 2015). Activation of GPR81 triggers Gi-mediated pathway that in turn inhibits Adenylate cyclase (AC), resulting in a decrease in cyclic AMP (cAMP) levels and changes in numerous intracellular mechanisms (Ahmed et al., 2009, 2010).

In a key study by Suzuki et al. (2011), transfer of lactate from astrocytes to neurons was found to be critical to mediate synaptic plasticity and memory consolidation. Pharmacological inhibition or genetic targeting of MCT2 irreversibly impairs long-term memory in mice (Newman et al., 2011; Suzuki et al., 2011). Long-term memory impairment could be reversed in MCT4-deficient mice by intrahippocampal administration of lactate, but not glucose (Suzuki et al., 2011). These results indicate that the neuronal uptake of lactate is important for the establishment of long-term memories. Further, degradation of glycogen, which, in the brain, is exclusively localized in astrocytes, is required for memory formation (Newman et al., 2011; Suzuki et al., 2011). Interestingly, exercise-mediated lactate increase was shown to enhance lactate levels in the hippocampus and to be beneficial for memory in mice (El Hayek et al., 2019). Activation of astrocytic, but not neuronal, β2-adrenergic receptors led to lactate production that mediated memory formation (Gao et al., 2016; Dong et al., 2017). Furthermore, lactate mediates neuroprotective effects following traumatic brain injury (TBI) (Alvarez et al., 2014; Zhou et al., 2018), hypoxia (Schurr et al., 1997), cerebral ischemia, and glutamate-mediated excitotoxicity (Bliss et al., 2004; Berthet et al., 2009; Jourdain et al., 2016) and was found to promote adult hippocampal neurogenesis (Lev-Vachnish et al., 2019). Interestingly, lactate has a dual impact on NMDA receptors. With low glutamate, lactate stimulates NMDA receptor signaling, resulting in plasticity gene induction and memory consolidation. However, in excitotoxic conditions with high glutamate, lactate decreases NMDA receptor-mediated signaling, thereby preventing glutamate-induced neuronal death (Jourdain et al., 2018). Recent evidence *in vivo* indicates that lactate is preferred to glucose as an energy substrate in active neurons, and that lactate metabolism shapes neuronal activity through K_*ATP*_ channels (Karagiannis et al., 2021). An important study finally showed that the effect of circulating glucose on neuronal depolarization was exclusively mediated by astrocyte-mediated lactate release, providing strong evidence for the role of ANLS *in vivo* (Sada et al., 2015).

## Brain Energy Metabolic Dysfunctions in Aging and Neurodegenerative Diseases

Aging leads to many physiological changes in body functioning, including cerebral and cognitive functions such as decreased working, spatial and episodic memory (Mattay et al., 2006; Glisky, 2007). With age, aerobic glycolysis and consumption of glucose were found to be severely decreased in the brain, particularly in the temporal, parietal and frontal lobes, and motor cortex (Goyal et al., 2017). This is accompanied in normal aging by the degeneration of brain structures, leading to loss in brain weight and volume (Dekaban, 1978; Fox and Schott, 2004), cortical thickness in the prefrontal cortex, medial temporal lobe and hippocampus (Sowell et al., 2003; Salat et al., 2004), gray matter atrophy, disruptions of white matter integrity (Bi et al., 2021), and synaptic density (Masliah et al., 1993). These non-pathological changes contribute to age-related cognitive decline in elderly subjects (Resnick et al., 2003; Yang et al., 2015; Bender et al., 2016).

Advances in neuroimaging techniques, such as MRI and PET allow to investigate the dynamic brain changes with aging *in vivo*. For instance, brain network connectome, which is assessed through diffusion MRI tractography efficiency, was found to decline with age in specific brain regions such as the hippocampus, thalamus, and frontal and parietal cortices (Bi et al., 2021). Glucose hypometabolism was observed with aging in the anterior cingulate cortex, several parts of the orbital and frontal gyrus and in the thalamus (Bi et al., 2021). By combining these two measurements, the study revealed a close coupling between age-dependent decreased brain network connectome and hypometabolism in specific brain regions that include frontal and temporal lobes, cingulate gyrus, hippocampus and hypothalamus (Gong et al., 2009; Bi et al., 2021). Importantly, several studies showed that glucose hypometabolism is due to a decrease of brain aerobic glycolysis as measured by the difference between glucose and oxygen consumptions (Goyal et al., 2017; Hipkiss, 2019; Tang, 2020; Yan et al., 2020). Both animal models and human studies showed that aging is characterized by a decreased aerobic glycolysis in astrocytes (Goyal et al., 2017) and mitochondrial oxidative phosphorylation in neurons (Boumezbeur et al., 2010; Jiang and Cadenas, 2014). It has been proposed that pathological neurons first exhibit mitochondrial dysfunction and compensatory increase in oxidative phosphorylation that results in a competition for a limited energetic resource, i.e., astrocyte-derived lactate, as the fuel of oxidative phosphorylation (Demetrius and Driver, 2015). This competition for energetic resource leads to deleterious consequences on initially healthy neurons in the vicinity of neurons with mitochondrial dysfunction, thereby spreading neurodegeneration and development of the pathological state, from normal aging to neurodegeneration (Demetrius et al., 2014). At the cellular level, impaired glucose uptake is correlated with a decrease in the expression and membrane translocation of the insulin-sensitive neuronal glucose transporters, GLUT3 and GLUT4, which influence neuronal survival in the rat brain (Jiang et al., 2013). Decrease of microvascular endothelium GLUT1 was also observed in the hypometabolic rat brain (Jiang et al., 2013). The disruption in glucose metabolism due to the loss of glucose transporters is closely associated with synaptic dysfunction and renders neurons vulnerable to degeneration.

Alzheimer’s disease, the most prevalent cause of age-associated dementia, is a progressive neurodegenerative disease with biochemical, metabolic and physiological changes that impact memory, thinking and behavior. In addition to the historical description of the pathology that include β amyloid plaques and hyperphosphorylated tau in the brain, it is characterized by clear mitochondrial and metabolic impairments (Butterfield and Halliwell, 2019). Hence, AD can be considered as a metabolic disease with impairment in mitochondrial bioenergetics, as well as glucose brain import and metabolism (Zulfiqar et al., 2019). Brain glucose hypometabolism appears early in the genesis of the pathology and is frequently present before the onset of clinically measurable symptoms (Costantini et al., 2008; Cunnane et al., 2011). For instance, numerous studies have highlighted reduced regional activity-dependent glucose uptake and utilization in AD using ^18^F-fluorodeoxyglucose (FDG) PET (Ferreira et al., 2010; Demetrius and Driver, 2013; Tomi et al., 2013; Demetrius et al., 2014; Fu and Jhamandas, 2014; Yin et al., 2016; Weise et al., 2018). These decreases are mostly observed in the parieto-temporal and posterior cingulate cortices and extended to the frontal areas while disease advances, whereas primary motor and visual cortices are less severely affected, and cerebellum, thalamus and basal ganglia are relatively spared (Friedland et al., 1985; Koss et al., 1985; Minoshima et al., 1997). In AD, degeneration occurs in the locus coeruleus (LC) depending on the disease progression (Chan-Palay and Asan, 1989; Rub et al., 2001; Wilson et al., 2013; Arendt et al., 2015; Peterson and Li, 2018). Noradrenaline (NA), which is released from the LC, activates cellular response in astrocytes that trigger increase in Ca^2+^ and cAMP, resulting in numerous cellular responses including enhanced aerobic glycolysis (Arendt et al., 2015; Vardjan et al., 2018). Therefore, early destruction of the LC may contribute, at least in part, to the impaired glucose metabolism in AD (Moore and Bloom, 1979). Another study reported a reduction of several glycolysis intermediates in the cerebrospinal fluid (CSF) of AD patients compared with controls (Bergau et al., 2019). In postmortem AD brains, dysregulation of nutrient transporters was observed, with a decrease of neuronal GLUT3 and astrocytic GLUT1 (Simpson et al., 1994; Harr et al., 1995; Mooradian et al., 1997). A similar reduction in GLUT1 and lactate transporters has been reported in culture of astrocytes from AD mouse model (Merlini et al., 2011). Postmortem studies of AD brains also revealed alterations in glycolytic enzymes activity, glucose utilization and amino acid metabolism (Marcus and Freedman, 1997; Palmer, 1999; Butterfield and Halliwell, 2019). Several genes involved in energy regulation were downregulated in AD patients and mouse model of AD (Liang et al., 2008). AD symptoms essentially never occur without glucose hypometabolism, and the extent of these metabolic changes are strongly correlated with the severity of clinical symptoms (Woo et al., 2010; Thomas et al., 2015). Of relevance, mitochondrial dysfunction, which is associated with age-related neurodegeneration, is also particularly important in AD (Beal, 2005; Yao and Brinton, 2011). In line with the decreased glycolysis in AD brains, an interesting recent study showed that levels of lactate were reduced in the CSF of patients with AD, although no correlation were found between CSF lactate and amyloid levels (Bonomi et al., 2021). Recently, physical exercise was found to have beneficial effects in AD through the improvement of brain glucose metabolism. Thus, aerobic exercise leads to the maintenance of brain glucose uptake in mild AD patients (Robinson et al., 2018), and to the protection against hypometabolism in brain regions particularly vulnerable in AD (Dougherty et al., 2017).

In humans, Apolipoprotein E (APOE) exists in three different isoforms: APOE2, APOE3 and APOE4. Homozygous and heterozygous carriers of APOE4 have respectively 12 fold and 2–3 fold times increased risk of developing late-stage AD than APOE2 or 3 carriers (Belloy et al., 2019). Depending on the ethnicity, 10–25% of the population is carrier of APOE4, which makes it the most prevalent genetic risk factor for AD. APOE4 has been clearly linked to brain hypometabolism, which was shown to precede neurodegeneration by years in APOE4(+) patients (Farrer et al., 1997; Raber et al., 2004; Reiman et al., 2004). APOE is a major cholesterol carrier involved in lipid metabolism. In the brain, APOE is primarily produced by astrocytes and regulates lipids delivery to neurons that are necessary for their structural maintenance, as well as injury repair (Xu et al., 1996, 2006; Mahley and Rall, 2000; Bu, 2009). In conditions of stress or injury, APOE can also be expressed by neurons (Mahley et al., 2006). Several studies have shown strong association between APOE4, metabolic genes expression and cerebral glucose uptake in human brains (Jagust et al., 2012; Carbonell et al., 2016; Wu et al., 2018). Mouse models carrying the APOE4 human allele also have reduced metabolic gene expression and cerebral glucose uptake compared to APOE3 expressing models (Alata et al., 2015; Lin et al., 2017; Williams et al., 2020). At the cellular level, APOE4-expressing astrocytes exhibit altered glycolysis, glucose uptake and lactate secretion (Wu et al., 2018; Williams et al., 2020). Interestingly, lactate transferred from astrocytes to neurons is used for the synthesis of lipid droplets in neurons, which in turn are transported back to astrocytes through carriers that include fatty acid transport proteins (FATPs) and apolipoproteins neurodegeneration (Liu et al., 2017). Expression of APOE4 impairs this transport of lipid droplets between neurons and astrocytes, which in turn promotes neurodegeneration (Liu et al., 2017).

Brain insulin resistance is also believed to contribute to metabolic dysfunctions in AD (Rivera et al., 2005). Thus, a growing body of epidemiological and molecular evidence indicates an overlap in risk, comorbidity, and pathophysiological mechanisms across Type 2 diabetes (T2D), mild cognitive impairment (MCI), AD and other types of dementia such as vascular dementia, Lewy body dementia (LBD) and FTD (Arnold et al., 2018). Studies also indicate that T2D patients are at increased risk of developing MCI or AD (Arnold et al., 2018). While insulin resistance is a central feature of T2D, research from the past few years has also shown that it is present in the brains of patients with dementia, even in the absence of T2D (De Felice et al., 2009; Zhao and Townsend, 2009; El Khoury et al., 2014). Moreover, cerebral levels of insulin and insulin receptor (IR) are lower in the brain of AD patients, and evidence for insulin signaling impairment in post-mortem brain tissue of AD patients and in animal models of AD has been shown (Steen et al., 2005; Chiu et al., 2008; Talbot et al., 2012). Insulin and insulin-like growth factors (IGFs) regulate key neuronal functions such as survival, energy metabolism and synaptic plasticity (Hoyer, 2002). Interestingly, insulin-mediated signaling pathways are impacted by APOE4 through the reduction of the expression of insulin receptor substrate 1 (IRS1) and Akt pathway in both mouse models and human brain tissue (Ong et al., 2014; Keeney et al., 2015), and the sequestration of IR in endosomes in an age-dependent manner (Zhao et al., 2017).

Human and animal studies have shown that dysregulation of insulin function contributes to aging and to the development of neurodegenerative diseases (Craft and Watson, 2004). In this context, impaired glucose utilization, mitochondrial dysfunction, reduced ATP production, and energy shortage in AD led to the hypothesis that these abnormalities could be mediated, at least in part, by desensitization of IR in the brain (Hoyer, 2002; Craft and Watson, 2004; de la Monte, 2009). Several preclinical studies have highlighted the impact of insulin dysregulation in models of cognition. In mice, intracerebroventricular injection of streptozotocin was found to reduce brain glucose metabolism, mitochondrial function, IR activity and spatial learning and memory (Duelli et al., 1994; Hoyer et al., 2000; de la Monte and Wands, 2006). Experimental induction of brain insulin resistance and insulin deficiency in mice causes AD-like neurodegeneration and cognitive impairment (Lester-Coll et al., 2006). In the brain, both neurons and astrocytes are impacted by insulin signaling. In neurons, insulin signaling modulates the expression of GABA, NMDA and AMPA receptors, catecholamine release, and glucose uptake via GLUT3. In astrocytes, insulin enhances glycogen storage, stimulates glucose uptake via GLUT1 and modulates inflammatory response (Heni et al., 2011; Arnold et al., 2018). Interestingly, activation of insulin-mediated pathways was downregulated in astrocytes in response to elevated chronic insulin levels, but not in neurons (Clarke et al., 1984). These cellular differences could have implications in the effects of T2D and insulin resistance on the function of different brain cell types.

In AD brains, reactive astrocytes are preferentially located in the vicinity of amyloid plaques, where they exhibit abnormal morphology (Rodriguez et al., 2009; Acosta et al., 2017; Liddelow et al., 2017). In the early stage of the disease, activated astrocytes have neuroprotective action by internalizing and degrading amyloid plaques, while upon progression of the disease, deposit of amyloid plaques leads to astrocytic death that in turn contribute to further development of the pathology (Nagele et al., 2004). Regarding the consequences of hypometabolic state in the brain, a study showed that amyloid plaques impair glucose uptake by interfering with exocytosis-dependent GLUT3 membrane expression (Uemura and Greenlee, 2001; Prapong et al., 2002). Several reports have described some adaptations of the astrocytic metabolism to amyloid plaques *in vitro*, with alterations of glycolysis and mitochondrial activity (Allaman et al., 2010; Oksanen et al., 2017; van Gijsel-Bonnello et al., 2017; Carter et al., 2019) and the activation of several intracellular cascades leading to inflammation, oxidative stress and calcium dysregulation (De Strooper and Karran, 2016).

## Current Therapeutic Strategies to Target Brain Hypometabolism

### Insulin Signaling

Considering the hypometabolic state and the emerging consideration of insulin signaling in AD, a number of therapeutic strategies targeting insulin-mediated pathways have been considered in order to restore brain energy metabolism (Kellar and Craft, 2020). These approaches include the use of insulin sensitizer agents or intranasal insulin to restore insulin signaling in AD, as well as antidiabetic drugs such as Metformin and Glucagon-like peptide-1 receptor (GLP-1R) agonists.

First, intranasal insulin has been developed with the objective to efficiently deliver insulin directly into the brain without changing peripheral levels that could cause insulin resistance (Born et al., 2002). Insulin has been known for many years to positively modulate brain glucose utilization (Havrankova et al., 1978; Bingham et al., 2002; Taouis and Torres-Aleman, 2019). In animal models of AD, intranasal insulin was found to reduce cerebral oxidative stress, tau phosphorylation and amyloid load, and improves cognitive functions (Barone et al., 2019) (see Table 1). In humans, intranasal insulin has shown promising clinical data in MCI and AD (Kellar and Craft, 2020) (see Table 2). For instance, a pilot trial reported improvement of cognition in healthy volunteers after intranasal insulin administration (Benedict et al., 2004, 2007). A subsequent study confirmed positive effect of intranasal insulin in patients with MCI or mild AD (Reger et al., 2008a,b). Further study on over 100 patients with MCI or mild to moderate AD reported some preservation of cognition and function, and higher cerebral glucose utilization assessed by FDG PET, although no changes were observed in AD biomarkers (Craft et al., 2012; Claxton et al., 2013). These results led to the establishment of a larger Phase 2 and 3 studies that have enrolled nearly 300 people with MCI or early-stage AD. In this trial, treatment with intranasal insulin showed positive impact on primary outcome ADAS-Cog12 memory assessment at 12 and 18 months in a patient’s cohort that has used one of the two devices used for insulin delivery, while another patient’s cohort that has used another delivery device failed to benefit from the treatment (Craft et al., 2020). Other series of clinical studies have evaluated long-acting insulin analog Detemir and showed that treatment of 50 MCI or AD patients led to memory improvement in APOE4 carriers, but worsened memory in non-carriers (Claxton et al., 2013). Another study showed that insulin, but not long-acting analog Detemir, increased memory and preserved volume in several brain regions (Craft et al., 2017). A small trial examining the effect of the rapid acting insulin analog Glulisine in patients with MCI or middle-stage AD failed to show any acute impact in cognition (Rosenbloom et al., 2021). Nasal insulin has also been tested in other neurodegenerative diseases, showing for example improved clinical outcome in PD severity (Novak et al., 2019).

**TABLE 1:** Preclinical evidence of compounds targeting brain energy metabolism.

Treatment	Model	Results	References
**Insulin signaling**			
Insulin	Human primary astrocytes	↑ Glucose uptake and glycogen storage in astrocytes	Heni et al., 2011; Arnold et al., 2018
Insulin (intranasal)	Aged APP/PS1/Tau (3Tg) AD mouse models	↑ Cognition; ↓ Cerebral oxidative stress, tau phosphorylation, Aβ load	Barone et al., 2019
**GLP1-R agonists**			
Liraglutide	APP/PS1, 3Tg, Aβ_1–42_ ICV injections AD mouse model	↑ Neuronal survival, synaptic function, learning and memory ↓ Neuroinflammation, amyloid plaque, hyperphosphorylated Tau	McClean et al., 2011, 2015; Hansen et al., 2016a,b; Qi et al., 2016; Chen et al., 2017
Liraglutide	Aβ cortical injection in non-human primate	↑ Insulin signaling, synapse number; ↓ Neuroinflammation	Lourenco et al., 2013; Batista et al., 2018
Liraglutide	5 × FAD AD mouse model	Restored defective metabolism of astrocytes (incl. lactate release); ↑ Neuroprotection through enhanced astrocytic glycolysis; ↑Cognition	Zheng et al., 2021
Liraglutide	Rodent models of PD, stroke and TBI	↑ Neuroprotection and behavioral activity in mouse and rat models of PD; ↑ Brain repair after cerebral ischemic injury; ↑ Cognition; ↓ neurodegeneration and neuroinflammation in mouse and rat models of TBI	Liu et al., 2015; Hansen et al., 2016b; Badawi et al., 2017; Bader et al., 2019; He et al., 2020
Exendin-4	3Tg AD + STZ-induced-T2D mouse model	↑ Plasma insulin levels; ↑ Aβ brain levels	Li et al., 2010
Exendin-4	Mouse and rat models of PD and TBI	↑ Neuroprotection, adult neurogenesis, behavioral activity	Perry and Greig, 2005; Perry et al., 2007; Bertilsson et al., 2008; Harkavyi et al., 2008; Li et al., 2009; Eakin et al., 2013; Rachmany et al., 2013
**Metformin**			
Metformin	Primary rat astrocytes	↑ Glycolysis and lactate production by astrocytes	Westhaus et al., 2017
Metformin	High fat diet in mice and rats	↑ Mitochondrial function, neuroprotection, cognition, autophagy in mouse models. One study showed no effect on cognition in rats (McNeilly et al., 2012)	McNeilly et al., 2012; Pintana et al., 2012; Lennox et al., 2014; Allard et al., 2016; Chen et al., 2021
**Ketogenic diet**			
KD	Rat	↑ Brain GLUT1 and MCT1 levels	Leino et al., 2001
KD	Mouse	↑ Brain mitochondrial function, ATP levels, oxidative stress resistance	Sullivan et al., 2004
KD, ketone ester	APP, APP/PS1, 3Tg AD mouse models	↑ Glycolysis, mitochondrial functions, cognition, motor performance ↓ Anxiety, Aβ levels, hyperphosphorylated Tau	Van der Auwera et al., 2005; Beckett et al., 2013; Kashiwaya et al., 2013; Zhang et al., 2013; Pawlosky et al., 2017
β-HB	MPTM-induced mouse model of PD	↑ Mitochondrial function, motor performance	Tieu et al., 2003
Caprylic triglyceride	SOD1-G93A mouse model of ALS	↑ Neuroprotection, motor performance	Zhao et al., 2012

*3Tg, triple transgenic; PS1, presenilin 1; Aβ, Amyloid β; AD, Alzheimer’s disease; ALS, Amyotrophic lateral sclerosis; APP, amyloid precursor protein; β-HB, β-hydroxybutyrate; GLUT1, glucose transporter 1; ICV, intracerebroventricular; GLP-1R, Glucagon-like peptide-1 receptor; KD, ketogenic diet; MCT1, monocarboxylate transporter 1; PD, Parkinson’s disease; STZ, Streptozotocin; T2D, Type 2 Diabetes.*

**TABLE 2:** Clinical evidence of compounds targeting brain energy metabolism in neurodegenerative diseases.

Treatment	Indication	Study design	Results	References
**Insulin**				
Intranasal insulin	Healthy	Intranasal insulin (4 × 40 IU/d) vs. placebo; 8 weeks; 38 subjects	↑ Declarative memory (delayed recall of words) and mood; No changes in blood glucose and plasma insulin	Benedict et al., 2004
Intranasal insulin or ASP-I	Healthy	Acute/8 weeks intranasal insulin or ASP-I (rapid acting insulin analog) (4 × 40IU/day) vs. placebo; 36 male subjects	↑ Declarative memory (word lists) after long-term administration (ASP-I > insulin); No change in blood glucose and plasma insulin	Benedict et al., 2007
Intranasal insulin	MCI or AD	Acute intranasal insulin (10, 20, 40 or 60 IU) vs. placebo; 33 patients	↑ Verbal memory in APOE4 (–) patients (max at 20 IU) ↓ Verbal memory in APOE4 (+) patients (n.s.); No change in blood insulin and glucose levels	Reger et al., 2008a
Intranasal insulin	Early AD	Intranasal insulin (20 IU BID) vs. placebo; 21 days; 24 patients	↑ Verbal information retention after delay, attention, functional status; ↑ Aβ40/42 ratio; No change in blood insulin and glucose levels	Reger et al., 2008b
Intranasal insulin	MCI or mild to moderate AD	Intranasal insulin (20, 40 IU) vs. placebo; 4 months; 104 patients	↑ Memory (delayed story) (ADAS-Cog and ADCS-ADL in younger participants); ↓ Dementia Severity Rating Scale; ↓ CMRGlc decline (FDG PET in precuneus, frontal and occipital cortices)	Craft et al., 2012
Intranasal insulin	MCI or AD	Intranasal insulin (20, 40 IU) vs. placebo; 4 months; 104 subjects	↑ Memory (delayed story; dose and sex-dependent); No change in memory in APOE4 (+) subjects	Claxton et al., 2013
Intranasal insulin or detemir	MCI or mild to moderate AD	Intranasal insulin, insulin analog detemir or placebo; 4 months; 36 patients	↑ Memory composite (delayed list and story recall) and preserved brain volume after insulin (not detemir); ↓ CSF Tau-P181/Aβ42 after insulin (not detemir); No change in daily functioning (insulin or detemir)	Craft et al., 2017
Intranasal insulin	PD	Intranasal insulin (40 IU) vs. placebo; 4 weeks; 16 patients	↑ Cognition (verbal fluency) and motor function	Novak et al., 2019
Intranasal insulin	MCI or AD	Intranasal insulin (40 IU) vs. placebo; 12 months (followed by 6 months open label extension); 289 patients	No change in memory (ADAS-Cog-12) (differences between groups depending on the injection device used); No change in CSF AD biomarkers, CSF insulin or blood glucose	Craft et al., 2020
Intranasal glulisine	MCI or mild AD	Intranasal Glulisine (rapid-acting insulin analog) (20 IU BID) vs. placebo; 6 months; 35 patients	No change in cognition (ADAS-Cog13), CDR global score, FAQ or mood. No change in blood glucose or insulin levels	Rosenbloom et al., 2021
**GLP1-R agonists**				
Liraglutide	AD	Liraglutide vs. placebo; 6 months; 38 patients	↓ CMRGlc decline (FDG PET in precuneus, cerebellum, temporal and occipital cortices); No change in cognition or Aβ (global and regional brain areas)	Egefjord et al., 2012; Gejl et al., 2016
Liraglutide	MCI	Liraglutide vs. placebo; 12 weeks; 41 patients	↑ Connectivity in the DMN (fMRI); No change in cognition	Watson et al., 2019
Liraglutide	Mild AD	Liraglutide vs. placebo; 1 year; 204 patients (without T2D)	↑ Memory (composite *z*-score); ↑ Temporal lobe and total gray matter volumes; No change in CMRGlc (FDG PET)	Femminella et al., 2019
Liraglutide	T2D	Liraglutide vs. placebo; 3 weeks; 40 patients (obesity with pre-diabetes or early-stage T2D)	↑ Memory (composite z-score: attention, memory, executive control)	Vadini et al., 2020
Semaglutide	MCI or mild AD	Semaglutide vs. placebo; 2 years; 2 studies of 1840 patients	Estimated study completion date: 2025	Clinical trials NCT04777396 and NCT04777409
**Metformin**				
Metformin	AD	Long-term use of Metformin on 7’686 patients aged 65+	↑ Risk of developing AD with long-term use of Metformin (presumably through Vit B12 deficiency)	Imfeld et al., 2012
Metformin	MCI	Metformin vs. placebo; 1 year; 80 patients (overweight and non-diabetic)	↑ Memory on SRT; No change in ADAS-Cog, glucose uptake or plasma Aβ	Luchsinger et al., 2016
Metformin	MCI or AD	Metformin vs. placebo; 8 weeks; 20 patients (non-diabetic)	↑ Executive functions; ↑ Learning and memory (n.s.); No change in AD biomarkers	Koenig et al., 2017
Metformin	AD	Meta analyses	↓ Dementia incidence in diabetic patients treated with Metformin	Campbell et al., 2018; Chin-Hsiao, 2019; Samaras et al., 2020; Sluggett et al., 2020
Metformin	MCI	Metformin vs. placebo; 2 years; 370 patients (overweight/obese w/o T2D)	Estimated study completion: 2025	Clinical trial NCT04098666
**Ketogenic Diet**				
MCT	Mild to moderate AD	MCT (Ketasyn/AC-1202) vs. placebo; 12 weeks; 152 patients	↑ Memory (ADAS-Cog) in APOE4(–), but not in APOE4(+) subjects	Henderson et al., 2009; Henderson and Poirier, 2011
KD	MCI	Low carbohydrates (5–10% cal.) vs. high carbohydrate (50% cal.) diet; 6 weeks; 23 patients	↑ Memory, positively correlated with ketone levels	Krikorian et al., 2012
MAD	MCI or early-stage AD	MAD vs. recommended diet; 12 weeks; 27 patients	↑ Episodic memory (n.s.); Low adherence	Brandt et al., 2019
KD	MCI in PD	KD vs. recommended diet; 8 weeks; 14 patients	↑ Memory, positively correlated with body weight loss; No effect on motor function	Krikorian et al., 2019
MCT	MCI	MCT (kMCT drink) vs. placebo drink; 6 months; 52 patients	↑ Cognitive functions	Fortier et al., 2019
MCT	Mild to moderate AD APOE4(–)	MCT (jelly) vs. placebo; 30 days; 46 patients	↑ Memory (ADAS-Cog)	Xu et al., 2020
MCT	Mild to moderate AD APOE4 (–)	MCT (Tricaprilin/AC-1204) vs. placebo; 26 weeks; 413 patients	No effect on memory (ADAS-Cog11)	Henderson et al., 2020
MCT	MCI	MCT (kMCT drink) vs. placebo drink; 6 months; 122 patients	↑ Cognitive functions	Fortier et al., 2021
MCT	Mild to moderate AD APOE4 (–)	MCT (AC-SD-03/CER-0001) vs. placebo; 26 weeks; 300 patients with decreased FDG PET signal	Estimated study completion: 2024	Clinical trial NCT04187547

*ADAS-Cog, The Alzheimer’s Disease Assessment Scale–Cognitive Subscale; AD, Alzheimer’s disease; ADCS-ADL, Alzheimer’s disease cooperative study – Activity of daily living; ALS, Amyotrophic lateral sclerosis; APOE4, apolipoprotein 4; BID, twice a day; CMRGlc, cerebral metabolic rate of glucose; CDR, clinical dementia rating; CSF, cerebrospinal fluid; DMN, default mode network; FAQ, Functional Activities Questionnaire; FDG-PET, fluorodeoxyglucose-positron emission tomography; IU, international units; KD, ketogenic diet; MAD, Modified Atkins Diet; MCI, mild cognitive impairment; MCT, Medium Chain Triglyceride; n.s., not significant; PD, Parkinson’s disease; SRT, Selective Remining Test; T2D, Type 2 Diabetes.*

### Glucagon-Like Peptide-1 Receptor Agonists

Another therapeutic strategy aiming at restoring brain metabolism by targeting insulin-related pathway is the use of Glucagon-Like Peptide-1 Receptor (GLP-1R) agonists. GLP-1 is an incretin hormone derived from proglucagon and secreted by the small intestine in response to food intake. GLP-1R is expressed in pancreatic β-cells, kidney, heart, and CNS (Yildirim Simsir et al., 2018). Activation of GLP-1R leads to insulin release by β-cells, which in turn stimulates glucose uptake. While GLP-1 is well known for its action in the regulation of peripheral metabolism, it was also shown to play key roles in CNS functions. For instance, GLP-1 is secreted by neurons in the nucleus tractus solitarius (NTS), which results in anorexic effect and transmit vagal motor information to the pancreas (Yildirim Simsir et al., 2018). Other studies showed that overexpression of GLP-1R in the rat hippocampus improves learning, memory and neuroprotection (During et al., 2003), while transgenic mice lacking GLP-1R have deficits in learning, synaptic plasticity and cognition (Abbas et al., 2009). Interestingly, effects of GLP-1 on energy balance were found to be mediated by astrocytes (Reiner et al., 2016). Thus, large number of astrocytes in the NTS respond to GLP-1R agonists by intracellular calcium and cAMP signaling, while blocking NTS astrocytes activity attenuated GLP-1R agonist effects on food intake in rats (Reiner et al., 2016). These data suggest that astrocytes play a role in the effects of GLP-1 in the brain (Cui et al., 2021).

Preclinical and clinical evidence indicate therapeutic potential for some of the GLP-1R agonists that are commonly used for the treatment of diabetes and obesity (Yildirim Simsir et al., 2018) (see Tables 1, 2, respectively). They include Liraglutide (Novo Nordisk), Semaglutide (Novo Nordisk) and Exendin-4, also known as Exenatide (AstraZeneca). First, in rodent models of AD, the GLP-1 analog Liraglutide was shown to promote neuronal survival, increase synaptic function, reduce neuroinflammation, amyloid plaque and hyperphosphorylated Tau, and support learning and memory (McClean et al., 2011, 2015; Hansen et al., 2016a; Qi et al., 2016; Chen et al., 2017; Holscher, 2018). In non-human primates, Liraglutide improved insulin signaling, reduced inflammation and restored synapse number that were caused by the cortical injections of β amyloid (Lourenco et al., 2013; Batista et al., 2018). Interestingly, a recent study using mouse model of AD showed that Liraglutide has a specific impact on astrocytes (Zheng et al., 2021). Treatment of AD mouse-derived astrocytes with Liraglutide resulted in a neuroprotective action and restored defective metabolic pathways, including lactate secretion (Zheng et al., 2021). Liraglutide also showed positive effects in preclinical models of other neurological diseases, including PD (Liu et al., 2015; Hansen et al., 2016b; Badawi et al., 2017), stroke (He et al., 2020) and TBI (Bader et al., 2019). The other GLP-1R agonist Exendin-4 also exhibited neuroprotective effects in a mouse model of AD (Li et al., 2010) and PD (Perry and Greig, 2005; Perry et al., 2007; Bertilsson et al., 2008; Harkavyi et al., 2008; Li et al., 2009; Eakin et al., 2013; Rachmany et al., 2013).

In humans, a pilot study has first tested the effects of 6-month Liraglutide treatment in 38 patients with AD (Egefjord et al., 2012). Liraglutide led to a clear increase in brain glucose utilization, as revealed by FDG PET (Femminella and Edison, 2014; Gejl et al., 2016). Another pilot trial was done with Liraglutide on 41 middle- to late-aged individuals with elevated blood glucose or diabetes and cognitive complaints. Primary outcome, which included fMRI before and after treatment, revealed improved connectivity within the default mode network (DMN), a system that is defective in AD (Watson et al., 2019). A study in 40 obese subjects with pre-diabetes or newly diagnosed T2D treated for 3 weeks with Liraglutide indicated an increase in short term memory (Vadini et al., 2020). Most recent Phase IIb study (ELAD study) on 204 patients with mild AD and no diabetes failed to see benefit of Liraglutide on primary outcome of FDG PET, but revealed improved in cognition, as well as temporal lobe and total brain gray matter volumes (Femminella et al., 2019). Semaglutide, another GLP-1R agonist, is set to be tested in two large Phase 3 studies (EVOKE and EVOKE Plus; NCT04777396 and NCT04777409). These trials each plan to enroll 1’840 patients with MCI or middle-stage dementia for a duration of 2 years. The clinical protocol is based on the *post hoc* data analyses from three clinical studies on T2D showing a 53% reduced risk of developing dementia in people who received Semaglutide or Liraglutide. Liraglutide, Semaglutide and Exendin-4 are also all currently being tested in clinical studies for PD.

### Metformin

Repositioning of Metformin, one of the most used medication for the treatment of T2D, has been another strategy to improve brain energy deficits in neurodegenerative diseases. Metformin acts through the activation of AMPK, an important regulator of glucose homeostasis. Activation of AMPK decreases gluconeogenesis, lowers blood glucose and restores insulin sensitivity. Metformin has been shown to reduce inflammation and oxidative stress, and to promote neurogenesis (Wang et al., 2012; Rotermund et al., 2018; Bharath et al., 2020). Interestingly, pleiotropic effects of Metformin have been hypothesized to be mediated, at least in part, by the increased circulating concentrations of lactate produced by AMPK-mediated glucose uptake, and its use as a direct energy source in various organs (Giaccari et al., 2021). In this context, Metformin was found to directly enhance glycolysis and production of lactate by astrocytes in the brain (Westhaus et al., 2017). Metformin was found to have positive impact in cognition in high fat diet mouse models (Pintana et al., 2012; Lennox et al., 2014; Allard et al., 2016), but others found no effect of Metformin in a rat model of high fat diet-induced cognitive deficit (McNeilly et al., 2012). In a mouse model of AD, Metformin attenuated Tau aggregates and amyloid load by ameliorating microglial autophagy (Chen et al., 2021) (Table 1). Interestingly, Metformin was found to repress the expression of Thioredoxin-interacting protein (Txnip), an inhibitor of Thioredoxin, through AMPK-mediated pathway (Chai et al., 2012). Repression of Txnip, which has been shown to improve glucose utilization and uptake (Parikh et al., 2007), is postulated to participate, at least in part, in the effects of Metformin on glucose metabolism.

In humans, several clinical studies have been assessing the impact of Metformin in MCI and AD (Table 2). First, a pilot study in 80 overweight non-diabetic people with MCI indicated that administration of Metformin for a duration of 1 year led to better performance on the Selective Reminding Test (SRT) of memory, but did not change other outcomes including ADAs-Cog, glucose uptake and plasma amyloid levels (Luchsinger et al., 2016). A smaller trial in 20 non-diabetic people with MCI or AD showed that Metformin improved executive functions, led to a trend toward better learning and memory, but did not change AD biomarkers (Koenig et al., 2017). However, contrasting study showed that long-term use of Metformin could also increase the risk of developing AD, presumably through its effect on Vitamin B12 deficiency, which is a cause of dementia (Imfeld et al., 2012). A multicentric Phase 2/3 trial that investigates the effect of Metformin on memory of 370 overweight or obese people with MCI is currently ongoing (NCT04098666). Interestingly, clinical meta-analyses showed that cognitive impairment, as well as dementia incidence, were significantly reduced in diabetic patients that were treated with Metformin (Campbell et al., 2018; Chin-Hsiao, 2019; Samaras et al., 2020; Sluggett et al., 2020).

### Ketogenic Diet

Ketogenesis is a physiological mechanism whereby ketone bodies (acetoacetate, β-hydroxybutyrate and acetone) are produced by the liver in response to fasting, exercise or reduced carbohydrate availability (Puchalska and Crawford, 2017). Ketone bodies that are released in the circulation can be used by extra-hepatic tissues as alternative energy sources. In the mitochondria of energy-consuming cells, ketone bodies are converted in acetyl-CoA and incorporated in the TCA cycle. The brain can adapt to the utilization of ketone bodies for up to 70% of its energy requirements (Veech, 2004). In humans, the classical ketogenic diet (KD) consists in a 4 to 1 ratio of fats to proteins and carbohydrates, which physiologically mimics the fasting state and leads to hepatic ketogenesis. Although safe and efficiently enhancing levels of circulating ketone bodies, KD usually lacks long-term adherence. Alternative diets consist in the less strict Modified Atkins Diet (MAD) or supplementation to the normal diet with exogenous ketogenic agents such as medium-chain triglycerides (MCTs).

Ketogenic diet is commonly used for the treatment of refractory epilepsy (D’Andrea Meira et al., 2019), and has been proposed as a therapeutic strategy to restore energy deficit in neuropathologies with metabolic dysfunction. Interestingly, transport of ketone bodies is directly affected by ketogenic diet through the upregulation of MCT1 and GLUT1 in the brain (Leino et al., 2001). Preclinical evidence showed promising results for the neuroprotective properties of KD (Paoli et al., 2014) (Table 1). For instance, KD in mice improved mitochondrial function, decreased oxidative stress, and increased ATP cerebral concentrations (Sullivan et al., 2004). In several transgenic mouse models of AD, KD was found to improve glycolysis, mitochondrial function and cognition, while reducing oxidative stress and amyloid deposition (Van der Auwera et al., 2005; Beckett et al., 2013; Kashiwaya et al., 2013; Zhang et al., 2013; Pawlosky et al., 2017). KD also improved metabolism, mitochondrial activity and motor functions in mouse models of PD (Tieu et al., 2003) and ALS (Zhao et al., 2012).

In humans, the impact of KD in neurodegenerative diseases has been tested in different clinical settings (Dewsbury et al., 2021) (Table 2). First, pilot study showed that 12-week KD in MCI or early-stage AD patients led to non-significant trend of memory improvement (Brandt et al., 2019). In a group of 23 patients with MCI, low carbohydrate diet was found to improve learning and memory performance compared to high carbohydrate diet (Krikorian et al., 2012). In a pilot study on 14 participants with MCI in PD, 8-week long KD enhanced cognitive performance (Krikorian et al., 2019). A larger multicentric study with 152 AD patients showed that 12-week-long administration of MCT Ketasyn/AC-1202 (Cerecin), improved memory performance in APOE4(–), but not in APOE4(+) patients (Henderson et al., 2009; Henderson and Poirier, 2011). Another pilot study showed that treatment with MCTs in APOE4(–) AD patients for 30 days improved memory performance (Xu et al., 2020). However, no significant differences were observed in subsequent trial on 413 AD patients after 26-week long intervention with MCT Tricaprilin/AC-1204 (Cerecin) (Henderson et al., 2020). Despite these contrasting results, Phase 3 trial with Cerecin’s MCT AC-SD-03/CER-0001 has been registered and plans to enroll 300 people with mild to moderate AD that exhibit decreased FDG PET signal and have APOE4(–) genotype (NCT04187547). Finally, a clinical study that assessed the metabolic effect of MCT-based regimen kMCT-ONS (Nestlé Health Science) in 52 people with MCI reported increase in plasma and brain ketones, while brain glucose uptake did not differ (Fortier et al., 2019). A larger 6-month trial in 122 MCI patients showed that treatment with kMCT-ONS diet led to improvements in memory tests, executive function and language (Fortier et al., 2021).

## Conclusion

**FIGURE 1:**
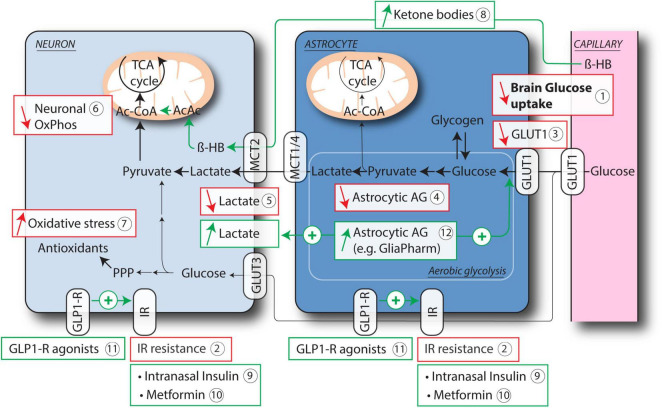
Age-related astrocytic and neuronal deficits leading to brain hypometabolism and current therapeutic strategies. Brain glucose hypometabolism is a hallmark of aging and neurodegeneration, as shown in particular by FDG PET studies (1). Resistance to Insulin has been proposed to account, at least in part, for this hypometabolism (2). Astrocytes and neurons both express insulin receptor (IR). Other key features of brain hypometabolism include reduced expression of GLUT1 on astrocytes and endothelial cells (3), decreased aerobic glycolysis (AG) in astrocytes (4) and consequent impaired release of lactate (5), reduced mitochondrial activity in neurons (6) and increased oxidative stress (7). Therapeutic strategies that aim at restoring brain energy metabolism include the use of ketone bodies as alternative energy source for neuronal mitochondrial OxPhos, either through ketogenic diet or medium chain triglycerides (8), targeting IR resistance either directly with intranasal Insulin (9) or Metformin (10), or via activation of GLP-1R (11). Another specific therapeutic approach consists in improving astrocytic AG (12), which results in increased glucose uptake and lactate release by astrocytes. Ac-CoA, acetyl-CoA; AcAc, acetoacetate; β-HB, β-hydroxybutyrate; GLP1-R, glucagon-like peptide 1 receptor; GLUT1, 3, glucose transporter 1, 3; IR, insulin receptor; MCT1, 2, 4, monocarboxylate transporter 1, 2, 4; OxPhos, oxidative phosphorylation; PPP, pentose phosphate pathway; TCA cycle, tricarboxylic acid cycle.

Brain hypometabolism is one of the first homeostatic dysregulation that occurs in age-related neurodegenerative diseases. Aging, APOE4 and insulin resistance are among the key factors that lead to brain hypometabolism. Hypometabolic changes are characterized by decreases in brain glucose uptake, expression of glucose transporters, astrocytic aerobic glycolysis, lactate release, neuronal mitochondrial function and increased oxidative stress (Figure 1). This homeostatic imbalance results in an energy gap, which renders neurons more vulnerable to a variety of insults and also decreases network connectivity (de la Torre, 2008). While brain hypometabolism occurs in physiological aging, it represents a significant contributing factor to a number of neurodegenerative diseases such as AD (Thomas et al., 2015; Butterfield and Halliwell, 2019), ALS (Lee et al., 2012; Vandoorne et al., 2018), depression (Rajkowska and Stockmeier, 2013), multiple sclerosis (Saab et al., 2016), migraine (Gross et al., 2019), epilepsy (de Melo et al., 2021), TBI (Carteron et al., 2018), retinal degeneration (Ait-Ali et al., 2015), stroke (Berthet et al., 2012), or spinal cord injury (SCI) (Babetto et al., 2020; Li et al., 2020).

To target brain hypometabolism, several different therapeutic approaches that have shown promising results are presented in this review. For instance, intranasal insulin, which increases brain glucose uptake, was shown to improve cognition in AD and MCI patients. GLP-1R agonists and Metformin also improved glucose utilization and cognitive function in AD mouse models and patients. Ketogenic diet, another therapeutic strategy that aims at providing alternative source of energy to neurons, improves metabolic functions and cognition in preclinical models and human AD and MCI patients. These approaches have shown promising results, but lack selectivity to brain pathways. More targeted metabolic approaches constitute future avenues of development to tackle hypometabolic neurological diseases. Among these innovative approaches, our strategy at GliaPharm aims at specifically improving aerobic glycolysis in astrocytes, which results in the activation of the ANLS, increase in brain glucose uptake and release of lactate that is used by neurons as preferential energy source. This approach led to promising results in the impact on brain energy metabolism and neuroprotection *in vitro* and in different preclinical models.

The increasing amount of evidence linking brain aging, neurological diseases and hypometabolism has therefore opened avenue for innovative therapeutic strategies, either through non-specific drug repurposing or targeted approaches to improve brain metabolism. These approaches could have disease-modifying impact in the management of the brain energy crisis in a number of neurological diseases.

## Author Contributions

All authors listed have made a substantial, direct, and intellectual contribution to the work, and approved it for publication.

## Conflict of Interest

EB, SL, SD, and CF were employed by company GliaPharm SA. PM is an advisor for GliaPharm SA.

## Publisher’s Note

All claims expressed in this article are solely those of the authors and do not necessarily represent those of their affiliated organizations, or those of the publisher, the editors and the reviewers. Any product that may be evaluated in this article, or claim that may be made by its manufacturer, is not guaranteed or endorsed by the publisher.

## References

[B1] Abbas T. Faivre E. Holscher C. (2009). Impairment of synaptic plasticity and memory formation in GLP-1 receptor KO mice: interaction between type 2 diabetes and Alzheimer’s disease. *Behav. Brain Res.* 205 265–271. 10.1016/j.bbr.2009.06.035 19573562

[B2] Acosta C. Anderson H. D. Anderson C. M. (2017). Astrocyte dysfunction in Alzheimer disease. *J. Neurosci. Res.* 95 2430–2447. 28467650 10.1002/jnr.24075

[B3] Ahmed K. Tunaru S. Offermanns S. (2009). GPR109A, GPR109B and GPR81, a family of hydroxy-carboxylic acid receptors. *Trends Pharmacol. Sci.* 30 557–562. 10.1016/j.tips.2009.09.001 19837462

[B4] Ahmed K. Tunaru S. Tang C. Muller M. Gille A. Sassmann A. (2010). An autocrine lactate loop mediates insulin-dependent inhibition of lipolysis through GPR81. *Cell Metab.* 11 311–319. 10.1016/j.cmet.2010.02.012 20374963

[B5] Ait-Ali N. Fridlich R. Millet-Puel G. Clerin E. Delalande F. Jaillard C. (2015). Rod-derived cone viability factor promotes cone survival by stimulating aerobic glycolysis. *Cell* 161 817–832. 10.1016/j.cell.2015.03.023 25957687

[B6] Alata W. Ye Y. St-Amour I. Vandal M. Calon F. (2015). Human apolipoprotein E varepsilon4 expression impairs cerebral vascularization and blood-brain barrier function in mice. *J. Cereb. Blood Flow Metab.* 35 86–94. 10.1038/jcbfm.2014.172 25335802 PMC4296574

[B7] Allaman I. Gavillet M. Belanger M. Laroche T. Viertl D. Lashuel H. A. (2010). Amyloid-beta aggregates cause alterations of astrocytic metabolic phenotype: impact on neuronal viability. *J. Neurosci.* 30 3326–3338. 10.1523/JNEUROSCI.5098-09.2010 20203192 PMC6634099

[B8] Allard J. S. Perez E. J. Fukui K. Carpenter P. Ingram D. K. De Cabo R. (2016). Prolonged metformin treatment leads to reduced transcription of Nrf2 and neurotrophic factors without cognitive impairment in older C57BL/6J mice. *Behav. Brain Res.* 301 1–9. 10.1016/j.bbr.2015.12.012 26698400 PMC4823003

[B9] Alle H. Roth A. Geiger J. R. (2009). Energy-efficient action potentials in hippocampal mossy fibers. *Science* 325 1405–1408. 10.1126/science.1174331 19745156

[B10] Alvarez Z. Castano O. Castells A. A. Mateos-Timoneda M. A. Planell J. A. Engel E. (2014). Neurogenesis and vascularization of the damaged brain using a lactate-releasing biomimetic scaffold. *Biomaterials* 35 4769–4781. 10.1016/j.biomaterials.2014.02.051 24636215

[B11] Arendt T. Bruckner M. K. Morawski M. Jager C. Gertz H. J. (2015). Early neurone loss in Alzheimer’s disease: cortical or subcortical? *Acta Neuropathol. Commun.* 3:10. 10.1186/s40478-015-0187-1 25853173 PMC4359478

[B12] Arnold S. E. Arvanitakis Z. Macauley-Rambach S. L. Koenig A. M. Wang H. Y. Ahima R. S. (2018). Brain insulin resistance in type 2 diabetes and Alzheimer disease: concepts and conundrums. *Nat. Rev. Neurol.* 14 168–181. 10.1038/nrneurol.2017.185 29377010 PMC6098968

[B13] Attwell D. Laughlin S. B. (2001). An energy budget for signaling in the grey matter of the brain. *J. Cereb. Blood Flow Metab.* 21 1133–1145. 10.1097/00004647-200110000-00001 11598490

[B14] Babetto E. Wong K. M. Beirowski B. (2020). A glycolytic shift in Schwann cells supports injured axons. *Nat. Neurosci.* 23 1215–1228. 32807950 10.1038/s41593-020-0689-4 PMC8758250

[B15] Badawi G. A. Abd El Fattah M. A. Zaki H. F. El Sayed M. I. (2017). Sitagliptin and liraglutide reversed nigrostriatal degeneration of rodent brain in rotenone-induced Parkinson’s disease. *Inflammopharmacology* 25 369–382. 10.1007/s10787-017-0331-6 28258522

[B16] Bader M. Li Y. Tweedie D. Shlobin N. A. Bernstein A. Rubovitch V. (2019). Neuroprotective effects and treatment potential of incretin mimetics in a murine model of mild traumatic brain injury. *Front. Cell Dev. Biol.* 7:356. 10.3389/fcell.2019.00356 PMC6965031 31998717

[B17] Bak L. K. Schousboe A. Waagepetersen H. S. (2006). The glutamate/GABA-glutamine cycle: aspects of transport, neurotransmitter homeostasis and ammonia transfer. *J. Neurochem.* 98 641–653. 10.1111/j.1471-4159.2006.03913.x 16787421

[B18] Barone E. Tramutola A. Triani F. Calcagnini S. Di Domenico F. Ripoli C. (2019). Biliverdin reductase-a mediates the beneficial effects of intranasal insulin in Alzheimer disease. *Mol. Neurobiol.* 56 2922–2943. 10.1007/s12035-018-1231-5 30073505

[B19] Barros L. F. San Martin A. Ruminot I. Sandoval P. Y. Baeza-Lehnert F. Arce-Molina R. (2020). Fluid brain glycolysis: limits, speed, location, moonlighting, and the fates of glycogen and lactate. *Neurochem. Res.* 45 1328–1334. 10.1007/s11064-020-03005-2 32144525

[B20] Batista A. F. Forny-Germano L. Clarke J. R. Lyra E. S. N. M. Brito-Moreira J. Boehnke S. E. (2018). The diabetes drug liraglutide reverses cognitive impairment in mice and attenuates insulin receptor and synaptic pathology in a non-human primate model of Alzheimer’s disease. *J. Pathol.* 245 85–100. 10.1002/path.5056 29435980 PMC5947670

[B21] Beal M. F. (2005). Oxidative damage as an early marker of Alzheimer’s disease and mild cognitive impairment. *Neurobiol. Aging* 26 585–586. 10.1016/j.neurobiolaging.2004.09.022 15708432

[B22] Beckett T. L. Studzinski C. M. Keller J. N. Paul Murphy M. Niedowicz D. M. (2013). A ketogenic diet improves motor performance but does not affect beta-amyloid levels in a mouse model of Alzheimer’s disease. *Brain Res.* 1505 61–67. 10.1016/j.brainres.2013.01.046 23415649 PMC3825515

[B23] Belanger M. Allaman I. Magistretti P. J. (2011). Brain energy metabolism: focus on astrocyte-neuron metabolic cooperation. *Cell Metab.* 14 724–738. 10.1016/j.cmet.2011.08.016 22152301

[B24] Belloy M. E. Napolioni V. Greicius M. D. (2019). A quarter century of APOE and Alzheimer’s disease: progress to date and the path forward. *Neuron* 101 820–838. 10.1016/j.neuron.2019.01.056 30844401 PMC6407643

[B25] Bender A. R. Prindle J. J. Brandmaier A. M. Raz N. (2016). White matter and memory in healthy adults: coupled changes over two years. *Neuroimage* 131 193–204. 10.1016/j.neuroimage.2015.10.085 26545457 PMC4848116

[B26] Benedict C. Hallschmid M. Hatke A. Schultes B. Fehm H. L. Born J. (2004). Intranasal insulin improves memory in humans. *Psychoneuroendocrinology* 29 1326–1334. 15288712 10.1016/j.psyneuen.2004.04.003

[B27] Benedict C. Hallschmid M. Schmitz K. Schultes B. Ratter F. Fehm H. L. (2007). Intranasal insulin improves memory in humans: superiority of insulin aspart. *Neuropsychopharmacology* 32 239–243. 10.1038/sj.npp.1301193 16936707

[B28] Bergau N. Maul S. Rujescu D. Simm A. Navarrete Santos A. (2019). Reduction of glycolysis intermediate concentrations in the cerebrospinal fluid of Alzheimer’s disease patients. *Front. Neurosci.* 13:871. 10.3389/fnins.2019.00871 PMC6713159 31496932

[B29] Bergersen L. Waerhaug O. Helm J. Thomas M. Laake P. Davies A. J. (2001). A novel postsynaptic density protein: the monocarboxylate transporter MCT2 is co-localized with delta-glutamate receptors in postsynaptic densities of parallel fiber-Purkinje cell synapses. *Exp. Brain Res.* 136 523–534. 10.1007/s002210000600 11291733

[B30] Bergersen L. H. Magistretti P. J. Pellerin L. (2005). Selective postsynaptic co-localization of MCT2 with AMPA receptor GluR2/3 subunits at excitatory synapses exhibiting AMPA receptor trafficking. *Cereb. Cortex* 15 361–370. 10.1093/cercor/bhh138 15749979

[B31] Berthet C. Castillo X. Magistretti P. J. Hirt L. (2012). New evidence of neuroprotection by lactate after transient focal cerebral ischaemia: extended benefit after intracerebroventricular injection and efficacy of intravenous administration. *Cerebrovasc. Dis.* 34 329–335. 23154656 10.1159/000343657

[B32] Berthet C. Lei H. Thevenet J. Gruetter R. Magistretti P. J. Hirt L. (2009). Neuroprotective role of lactate after cerebral ischemia. *J. Cereb. Blood Flow Metab.* 29 1780–1789. 10.1038/jcbfm.2009.97 19675565

[B33] Bertilsson G. Patrone C. Zachrisson O. Andersson A. Dannaeus K. Heidrich J. (2008). Peptide hormone exendin-4 stimulates subventricular zone neurogenesis in the adult rodent brain and induces recovery in an animal model of Parkinson’s disease. *J. Neurosci. Res.* 86 326–338. 10.1002/jnr.21483 17803225

[B34] Bharath L. P. Agrawal M. Mccambridge G. Nicholas D. A. Hasturk H. Liu J. (2020). Metformin enhances autophagy and normalizes mitochondrial function to alleviate aging-associated inflammation. *Cell Metab.* 32 44–55.e6. 10.1016/j.cmet.2020.04.015 32402267 PMC7217133

[B35] Bi Q. Wang W. Niu N. Li H. Wang Y. Huang W. (2021). Relationship between the disrupted topological efficiency of the structural brain connectome and glucose hypometabolism in normal aging. *Neuroimage* 226:117591. 10.1016/j.neuroimage.2020.117591 33248254

[B36] Bingham E. M. Hopkins D. Smith D. Pernet A. Hallett W. Reed L. (2002). The role of insulin in human brain glucose metabolism: an 18fluoro-deoxyglucose positron emission tomography study. *Diabetes* 51 3384–3390. 10.2337/diabetes.51.12.3384 12453890

[B37] Bittar P. G. Charnay Y. Pellerin L. Bouras C. Magistretti P. J. (1996). Selective distribution of lactate dehydrogenase isoenzymes in neurons and astrocytes of human brain. *J. Cereb. Blood Flow Metab.* 16 1079–1089. 10.1097/00004647-199611000-00001 8898679

[B38] Bliss T. M. Ip M. Cheng E. Minami M. Pellerin L. Magistretti P. (2004). Dual-gene, dual-cell type therapy against an excitotoxic insult by bolstering neuroenergetics. *J. Neurosci.* 24 6202–6208. 10.1523/JNEUROSCI.0805-04.2004 15240812 PMC6729663

[B39] Bolanos J. P. Peuchen S. Heales S. J. Land J. M. Clark J. B. (1994). Nitric oxide-mediated inhibition of the mitochondrial respiratory chain in cultured astrocytes. *J. Neurochem.* 63 910–916. 10.1046/j.1471-4159.1994.63030910.x 7519665

[B40] Bonomi C. G. De Lucia V. Mascolo A. P. Assogna M. Motta C. Scaricamazza E. (2021). Brain energy metabolism and neurodegeneration: hints from CSF lactate levels in dementias. *Neurobiol. Aging* 105 333–339. 10.1016/j.neurobiolaging.2021.05.011 34171631

[B41] Bonvento G. Sibson N. Pellerin L. (2002). Does glutamate image your thoughts? *Trends Neurosci.* 25 359–364. 10.1016/s0166-2236(02)02168-9 12079764

[B42] Born J. Lange T. Kern W. Mcgregor G. P. Bickel U. Fehm H. L. (2002). Sniffing neuropeptides: a transnasal approach to the human brain. *Nat. Neurosci.* 5 514–516. 10.1038/nn849 11992114

[B43] Bosshart P. D. Kalbermatter D. Bonetti S. Fotiadis D. (2019). Mechanistic basis of L-lactate transport in the SLC16 solute carrier family. *Nat. Commun.* 10:2649. 10.1038/s41467-019-10566-6 31201333 PMC6573034

[B44] Boumezbeur F. Mason G. F. De Graaf R. A. Behar K. L. Cline G. W. Shulman G. I. (2010). Altered brain mitochondrial metabolism in healthy aging as assessed by in vivo magnetic resonance spectroscopy. *J. Cereb. Blood Flow Metab.* 30 211–221. 10.1038/jcbfm.2009.197 19794401 PMC2949111

[B45] Brandt J. Buchholz A. Henry-Barron B. Vizthum D. Avramopoulos D. Cervenka M. C. (2019). Preliminary report on the feasibility and efficacy of the modified atkins diet for treatment of mild cognitive impairment and early Alzheimer’s disease. *J. Alzheimers Dis.* 68 969–981. 10.3233/JAD-180995 30856112

[B46] Brooks G. A. (2018). The science and translation of lactate shuttle theory. *Cell Metab.* 27 757–785. 10.1016/j.cmet.2018.03.008 29617642

[B47] Bu G. (2009). Apolipoprotein E and its receptors in Alzheimer’s disease: pathways, pathogenesis and therapy. *Nat. Rev. Neurosci.* 10 333–344. 10.1038/nrn2620 19339974 PMC2908393

[B48] Burda J. E. Bernstein A. M. Sofroniew M. V. (2016). Astrocyte roles in traumatic brain injury. *Exp Neurol* 275(Pt 3) 305–315. 10.1016/j.expneurol.2015.03.020 25828533 PMC4586307

[B49] Butterfield D. A. Halliwell B. (2019). Oxidative stress, dysfunctional glucose metabolism and Alzheimer disease. *Nat. Rev. Neurosci.* 20 148–160. 10.1038/s41583-019-0132-6 30737462 PMC9382875

[B50] Campbell J. M. Stephenson M. D. De Courten B. Chapman I. Bellman S. M. Aromataris E. (2018). Metformin use associated with reduced risk of dementia in patients with diabetes: a systematic review and meta-analysis. *J. Alzheimers Dis.* 65 1225–1236. 10.3233/JAD-180263 30149446 PMC6218120

[B51] Carbonell F. Zijdenbos A. P. Mclaren D. G. Iturria-Medina Y. Bedell B. J., Alzheimer’s Disease Neuroimaging Initiative. (2016). Modulation of glucose metabolism and metabolic connectivity by beta-amyloid. *J. Cereb. Blood Flow Metab.* 36 2058–2071. 10.1177/0271678X16654492 27301477 PMC5363668

[B52] Carter S. F. Chiotis K. Nordberg A. Rodriguez-Vieitez E. (2019). Longitudinal association between astrocyte function and glucose metabolism in autosomal dominant Alzheimer’s disease. *Eur. J. Nucl. Med. Mol. Imaging* 46 348–356. 10.1007/s00259-018-4217-7 30515545 PMC6333721

[B53] Carteron L. Solari D. Patet C. Quintard H. Miroz J. P. Bloch J. (2018). Hypertonic lactate to improve cerebral perfusion and glucose availability after acute brain injury. *Crit. Care Med.* 46 1649–1655. 10.1097/CCM.0000000000003274 29923931

[B54] Chai T. F. Hong S. Y. He H. Zheng L. Hagen T. Luo Y. (2012). A potential mechanism of metformin-mediated regulation of glucose homeostasis: inhibition of Thioredoxin-interacting protein (Txnip) gene expression. *Cell Signal.* 24 1700–1705. 10.1016/j.cellsig.2012.04.017 22561086

[B55] Chan-Palay V. Asan E. (1989). Alterations in catecholamine neurons of the locus coeruleus in senile dementia of the Alzheimer type and in Parkinson’s disease with and without dementia and depression. *J. Comp. Neurol.* 287 373–392. 10.1002/cne.902870308 2570794

[B56] Chen S. Sun J. Zhao G. Guo A. Chen Y. Fu R. (2017). Liraglutide improves water maze learning and memory performance while reduces hyperphosphorylation of tau and neurofilaments in APP/PS1/Tau triple transgenic mice. *Neurochem. Res.* 42 2326–2335. 10.1007/s11064-017-2250-8 28382596

[B57] Chen Y. Zhao S. Fan Z. Li Z. Zhu Y. Shen T. (2021). Metformin attenuates plaque-associated tau pathology and reduces amyloid-beta burden in APP/PS1 mice. *Alzheimers Res. Ther.* 13:40. 10.1186/s13195-020-00761-9 33563332 PMC7871393

[B58] Chin-Hsiao T. (2019). Metformin and the risk of dementia in type 2 diabetes patients. *Aging Dis.* 10 37–48. 30705766 10.14336/AD.2017.1202 PMC6345339

[B59] Chiu S. L. Chen C. M. Cline H. T. (2008). Insulin receptor signaling regulates synapse number, dendritic plasticity, and circuit function in vivo. *Neuron* 58 708–719. 10.1016/j.neuron.2008.04.014 18549783 PMC3057650

[B60] Chowdhury G. M. Jiang L. Rothman D. L. Behar K. L. (2014). The contribution of ketone bodies to basal and activity-dependent neuronal oxidation in vivo. *J. Cereb. Blood Flow Metab.* 34 1233–1242. 10.1038/jcbfm.2014.77 24780902 PMC4083391

[B61] Clarke D. W. Boyd F. T. Jr. Kappy M. S. Raizada M. K. (1984). Insulin binds to specific receptors and stimulates 2-deoxy-D-glucose uptake in cultured glial cells from rat brain. *J. Biol. Chem.* 259 11672–11675. 6384211

[B62] Claxton A. Baker L. D. Wilkinson C. W. Trittschuh E. H. Chapman D. Watson G. S. (2013). Sex and ApoE genotype differences in treatment response to two doses of intranasal insulin in adults with mild cognitive impairment or Alzheimer’s disease. *J. Alzheimers Dis.* 35 789–797. 10.3233/JAD-122308 23507773 PMC4144993

[B63] Costantini L. C. Barr L. J. Vogel J. L. Henderson S. T. (2008). Hypometabolism as a therapeutic target in Alzheimer’s disease. *BMC Neurosci.* 9(Suppl. 2):S16. 10.1186/1471-2202-9-S2-S16 PMC2604900 19090989

[B64] Craft S. Baker L. D. Montine T. J. Minoshima S. Watson G. S. Claxton A. (2012). Intranasal insulin therapy for Alzheimer disease and amnestic mild cognitive impairment: a pilot clinical trial. *Arch. Neurol.* 69 29–38. 21911655 10.1001/archneurol.2011.233 PMC3260944

[B65] Craft S. Claxton A. Baker L. D. Hanson A. J. Cholerton B. Trittschuh E. H. (2017). Effects of regular and long-acting insulin on cognition and Alzheimer’s disease biomarkers: a pilot clinical trial. *J. Alzheimers Dis.* 57 1325–1334. 10.3233/JAD-161256 28372335 PMC5409050

[B66] Craft S. Raman R. Chow T. W. Rafii M. S. Sun C. K. Rissman R. A. (2020). Safety, efficacy, and feasibility of intranasal insulin for the treatment of mild cognitive impairment and Alzheimer disease dementia: a randomized clinical trial. *JAMA Neurol.* 77 1099–1109. 10.1001/jamaneurol.2020.1840 32568367 PMC7309571

[B67] Craft S. Watson G. S. (2004). Insulin and neurodegenerative disease: shared and specific mechanisms. *Lancet Neurol.* 3 169–178. 10.1016/S1474-4422(04)00681-7 14980532

[B68] Cui Q. N. Stein L. M. Fortin S. M. Hayes M. R. (2021). The role of glia in the physiology and pharmacology of glucagon-like peptide-1: implications for obesity, diabetes, neurodegeneration and glaucoma. *Br. J. Pharmacol.* 10.1111/bph.15683 PMC8820182 34519040

[B69] Cunnane S. Nugent S. Roy M. Courchesne-Loyer A. Croteau E. Tremblay S. (2011). Brain fuel metabolism, aging, and Alzheimer’s disease. *Nutrition* 27 3–20. 10.1016/j.nut.2010.07.021 21035308 PMC3478067

[B70] Cureton E. L. Kwan R. O. Dozier K. C. Sadjadi J. Pal J. D. Victorino G. P. (2010). A different view of lactate in trauma patients: protecting the injured brain. *J. Surg. Res.* 159 468–473. 10.1016/j.jss.2009.04.020 19726055

[B71] D’Andrea Meira I. Romao T. T. Pires do Prado H. J. Kruger L. T. Pires M. E. P. Da Conceicao P. O. (2019). Ketogenic diet and epilepsy: what we know so far. *Front. Neurosci.* 13:5. 10.3389/fnins.2019.00005 PMC6361831 30760973

[B72] De Felice F. G. Vieira M. N. Bomfim T. R. Decker H. Velasco P. T. Lambert M. P. (2009). Protection of synapses against Alzheimer’s-linked toxins: insulin signaling prevents the pathogenic binding of Abeta oligomers. *Proc. Natl. Acad. Sci. U.S.A.* 106 1971–1976. 10.1073/pnas.0809158106 19188609 PMC2634809

[B73] de la Monte S. M. (2009). Insulin resistance and Alzheimer’s disease. *BMB Rep.* 42 475–481. 19712582 10.5483/bmbrep.2009.42.8.475 PMC4600067

[B74] de la Monte S. M. Wands J. R. (2006). Molecular indices of oxidative stress and mitochondrial dysfunction occur early and often progress with severity of Alzheimer’s disease. *J. Alzheimers Dis.* 9 167–181. 10.3233/jad-2006-9209 16873964

[B75] de la Torre J. C. (2008). Pathophysiology of neuronal energy crisis in Alzheimer’s disease. *Neurodegener. Dis.* 5 126–132. 10.1159/000113681 18322369

[B76] de Melo I. S. Pacheco A. L. D. Dos Santos Y. M. O. Figueiredo L. M. Nicacio D. Cardoso-Sousa L. (2021). Modulation of glucose availability and effects of hypo- and hyperglycemia on status epilepticus: what we do not know yet? *Mol. Neurobiol.* 58 505–519. 10.1007/s12035-020-02133-8 32975651

[B77] De Strooper B. Karran E. (2016). The cellular phase of Alzheimer’s disease. *Cell* 164 603–615. 26871627 10.1016/j.cell.2015.12.056

[B78] Dekaban A. S. (1978). Changes in brain weights during the span of human life: relation of brain weights to body heights and body weights. *Ann. Neurol.* 4 345–356. 10.1002/ana.410040410 727739

[B79] Demetrius L. A. Driver J. (2013). Alzheimer’s as a metabolic disease. *Biogerontology* 14 641–649. 10.1007/s10522-013-9479-7 24249045

[B80] Demetrius L. A. Driver J. A. (2015). Preventing Alzheimer’s disease by means of natural selection. *J. R. Soc. Interface* 12:20140919. 10.1098/rsif.2014.0919 25551134 PMC4277082

[B81] Demetrius L. A. Magistretti P. J. Pellerin L. (2014). Alzheimer’s disease: the amyloid hypothesis and the inverse warburg effect. *Front Physiol.* 5:522. 10.3389/fphys.2014.00522 PMC4294122 25642192

[B82] Dewsbury L. S. Lim C. K. Steiner G. Z. (2021). The efficacy of ketogenic therapies in the clinical management of people with neurodegenerative disease: a systematic review. *Adv. Nutr.* 12 1571–1593. 10.1093/advances/nmaa180 33621313 PMC8321843

[B83] Dong J. H. Wang Y. J. Cui M. Wang X. J. Zheng W. S. Ma M. L. (2017). Adaptive activation of a stress response pathway improves learning and memory through gs and beta-arrestin-1-regulated lactate metabolism. *Biol. Psychiatry* 81 654–670. 10.1016/j.biopsych.2016.09.025 27916196 PMC6088385

[B84] Dougherty R. J. Schultz S. A. Kirby T. K. Boots E. A. Oh J. M. Edwards D. (2017). Moderate physical activity is associated with cerebral glucose metabolism in adults at risk for Alzheimer’s disease. *J. Alzheimers Dis.* 58 1089–1097. 10.3233/JAD-161067 28527205 PMC5703045

[B85] Duelli R. Schrock H. Kuschinsky W. Hoyer S. (1994). Intracerebroventricular injection of streptozotocin induces discrete local changes in cerebral glucose utilization in rats. *Int. J. Dev. Neurosci.* 12 737–743. 10.1016/0736-5748(94)90053-1 7747600

[B86] During M. J. Cao L. Zuzga D. S. Francis J. S. Fitzsimons H. L. Jiao X. (2003). Glucagon-like peptide-1 receptor is involved in learning and neuroprotection. *Nat. Med.* 9 1173–1179. 10.1038/nm919 12925848

[B87] Eakin K. Li Y. Chiang Y. H. Hoffer B. J. Rosenheim H. Greig N. H. (2013). Exendin-4 ameliorates traumatic brain injury-induced cognitive impairment in rats. *PLoS One* 8:e82016. 10.1371/journal.pone.0082016 PMC3847068 24312624

[B88] Egefjord L. Gejl M. Moller A. Braendgaard H. Gottrup H. Antropova O. (2012). Effects of liraglutide on neurodegeneration, blood flow and cognition in Alzheimer’s disease - protocol for a controlled, randomized double-blinded trial. *Dan. Med. J.* 59:A4519. 23158895

[B89] El Hayek L. Khalifeh M. Zibara V. Abi Assaad R. Emmanuel N. Karnib N. (2019). Lactate mediates the effects of exercise on learning and memory through SIRT1-dependent activation of hippocampal brain-derived neurotrophic factor (BDNF). *J. Neurosci.* 39 2369–2382. 10.1523/JNEUROSCI.1661-18.2019 30692222 PMC6435829

[B90] El Khoury N. B. Gratuze M. Papon M. A. Bretteville A. Planel E. (2014). Insulin dysfunction and Tau pathology. *Front. Cell Neurosci.* 8:22. 10.3389/fncel.2014.00022 PMC3920186 24574966

[B91] Farrer L. A. Cupples L. A. Haines J. L. Hyman B. Kukull W. A. Mayeux R. (1997). Effects of age, sex, and ethnicity on the association between apolipoprotein E genotype and Alzheimer disease. A meta-analysis. APOE and Alzheimer disease meta analysis consortium. *JAMA* 278 1349–1356. 9343467

[B92] Femminella G. D. Edison P. (2014). Evaluation of neuroprotective effect of glucagon-like peptide 1 analogs using neuroimaging. *Alzheimers Dement.* 10 S55–S61. 10.1016/j.jalz.2013.12.012 24529526

[B93] Femminella G. D. Frangou E. Love S. B. Busza G. Holmes C. Ritchie C. (2019). Evaluating the effects of the novel GLP-1 analogue liraglutide in Alzheimer’s disease: study protocol for a randomised controlled trial (ELAD study). *Trials* 20:191. 10.1186/s13063-019-3259-x PMC6448216 30944040

[B94] Ferreira I. L. Resende R. Ferreiro E. Rego A. C. Pereira C. F. (2010). Multiple defects in energy metabolism in Alzheimer’s disease. *Curr. Drug Targets* 11 1193–1206. 10.2174/1389450111007011193 20840064

[B95] Figley C. R. Stroman P. W. (2011). The role(s) of astrocytes and astrocyte activity in neurometabolism, neurovascular coupling, and the production of functional neuroimaging signals. *Eur. J. Neurosci.* 33 577–588. 10.1111/j.1460-9568.2010.07584.x 21314846

[B96] Fortier M. Castellano C. A. Croteau E. Langlois F. Bocti C. St-Pierre V. (2019). A ketogenic drink improves brain energy and some measures of cognition in mild cognitive impairment. *Alzheimers Dement.* 15 625–634. 10.1016/j.jalz.2018.12.017 31027873

[B97] Fortier M. Castellano C. A. St-Pierre V. Myette-Cote E. Langlois F. Roy M. (2021). A ketogenic drink improves cognition in mild cognitive impairment: results of a 6-month RCT. *Alzheimers Dement.* 17 543–552. 10.1002/alz.12206 33103819 PMC8048678

[B98] Fox N. C. Schott J. M. (2004). Imaging cerebral atrophy: normal ageing to Alzheimer’s disease. *Lancet* 363 392–394. 10.1016/S0140-6736(04)15441-X 15074306

[B99] Friedland R. P. Brun A. Budinger T. F. (1985). Pathological and positron emission tomographic correlations in Alzheimer’s disease. *Lancet* 1:228. 10.1016/s0140-6736(85)92074-4 2857310

[B100] Fu W. Jhamandas J. H. (2014). Role of astrocytic glycolytic metabolism in Alzheimer’s disease pathogenesis. *Biogerontology* 15 579–586. 10.1007/s10522-014-9525-0 25106114

[B101] Gao V. Suzuki A. Magistretti P. J. Lengacher S. Pollonini G. Steinman M. Q. (2016). Astrocytic beta2-adrenergic receptors mediate hippocampal long-term memory consolidation. *Proc. Natl. Acad. Sci. U.S.A.* 113 8526–8531. 10.1073/pnas.1605063113 27402767 PMC4968707

[B102] Gejl M. Gjedde A. Egefjord L. Moller A. Hansen S. B. Vang K. (2016). In Alzheimer’s disease, 6-month treatment with GLP-1 analog prevents decline of brain glucose metabolism: randomized, placebo-controlled, double-blind clinical trial. *Front. Aging Neurosci.* 8:108. 10.3389/fnagi.2016.00108 PMC4877513 27252647

[B103] Giaccari A. Solini A. Frontoni S. Del Prato S. (2021). Metformin benefits: another example for alternative energy substrate mechanism? *Diabetes Care* 44 647–654. 10.2337/dc20-1964 33608326 PMC7896249

[B104] Glisky E. L. (2007). “Changes in cognitive function in human aging,” in *Brain Aging: Models, Methods, and Mechanisms*, ed. Riddle D. R. (Boca Raton, FL: CRC Press). 21204355

[B105] Gong G. Rosa-Neto P. Carbonell F. Chen Z. J. He Y. Evans A. C. (2009). Age- and gender-related differences in the cortical anatomical network. *J. Neurosci.* 29 15684–15693. 10.1523/JNEUROSCI.2308-09.2009 20016083 PMC2831804

[B106] Goyal M. S. Vlassenko A. G. Blazey T. M. Su Y. Couture L. E. Durbin T. J. (2017). Loss of brain aerobic glycolysis in normal human aging. *Cell Metab.* 26 353–360.e3. 10.1016/j.cmet.2017.07.010 28768174 PMC5573225

[B107] Gross E. C. Lisicki M. Fischer D. Sandor P. S. Schoenen J. (2019). The metabolic face of migraine - from pathophysiology to treatment. *Nat. Rev. Neurol.* 15 627–643. 10.1038/s41582-019-0255-4 31586135

[B108] Hansen H. H. Barkholt P. Fabricius K. Jelsing J. Terwel D. Pyke C. (2016a). The GLP-1 receptor agonist liraglutide reduces pathology-specific tau phosphorylation and improves motor function in a transgenic hTauP301L mouse model of tauopathy. *Brain Res.* 1634 158–170. 10.1016/j.brainres.2015.12.052 26746341

[B109] Hansen H. H. Fabricius K. Barkholt P. Mikkelsen J. D. Jelsing J. Pyke C. (2016b). Characterization of liraglutide, a glucagon-like peptide-1 (GLP-1) receptor agonist, in rat partial and full nigral 6-hydroxydopamine lesion models of Parkinson’s disease. *Brain Res.* 1646 354–365. 10.1016/j.brainres.2016.05.038 27233809

[B110] Harkavyi A. Abuirmeileh A. Lever R. Kingsbury A. E. Biggs C. S. Whitton P. S. (2008). Glucagon-like peptide 1 receptor stimulation reverses key deficits in distinct rodent models of Parkinson’s disease. *J. Neuroinflammation* 5:19. 10.1186/1742-2094-5-19 18492290 PMC2426681

[B111] Harr S. D. Simonian N. A. Hyman B. T. (1995). Functional alterations in Alzheimer’s disease: decreased glucose transporter 3 immunoreactivity in the perforant pathway terminal zone. *J. Neuropathol. Exp. Neurol.* 54 38–41. 7815078

[B112] Havrankova J. Schmechel D. Roth J. Brownstein M. (1978). Identification of insulin in rat brain. *Proc. Natl. Acad. Sci. U.S.A.* 75 5737–5741. 364489 10.1073/pnas.75.11.5737 PMC393044

[B113] He W. Wang H. Zhao C. Tian X. Li L. Wang H. (2020). Role of liraglutide in brain repair promotion through Sirt1-mediated mitochondrial improvement in stroke. *J. Cell Physiol.* 235 2986–3001. 10.1002/jcp.29204 31535381

[B114] Henderson S. T. Morimoto B. H. Cummings J. L. Farlow M. R. Walker J. (2020). A placebo-controlled, parallel-group, randomized clinical trial of AC-1204 in mild-to-moderate Alzheimer’s disease. *J. Alzheimers Dis.* 75 547–557. 10.3233/JAD-191302 32310169

[B115] Henderson S. T. Poirier J. (2011). Pharmacogenetic analysis of the effects of polymorphisms in APOE, IDE and IL1B on a ketone body based therapeutic on cognition in mild to moderate Alzheimer’s disease; a randomized, double-blind, placebo-controlled study. *BMC Med. Genet.* 12:137. 10.1186/1471-2350-12-137 PMC3213220 21992747

[B116] Henderson S. T. Vogel J. L. Barr L. J. Garvin F. Jones J. J. Costantini L. C. (2009). Study of the ketogenic agent AC-1202 in mild to moderate Alzheimer’s disease: a randomized, double-blind, placebo-controlled, multicenter trial. *Nutr. Metab. (Lond.).* 6:31. 10.1186/1743-7075-6-31 19664276 PMC2731764

[B117] Heni M. Hennige A. M. Peter A. Siegel-Axel D. Ordelheide A. M. Krebs N. (2011). Insulin promotes glycogen storage and cell proliferation in primary human astrocytes. *PLoS One* 6:e21594. 10.1371/journal.pone.0021594 PMC3124526 21738722

[B118] Herrero-Mendez A. Almeida A. Fernandez E. Maestre C. Moncada S. Bolanos J. P. (2009). The bioenergetic and antioxidant status of neurons is controlled by continuous degradation of a key glycolytic enzyme by APC/C-Cdh1. *Nat. Cell Biol.* 11 747–752. 10.1038/ncb1881 19448625

[B119] Herzig S. Raemy E. Montessuit S. Veuthey J. L. Zamboni N. Westermann B. (2012). Identification and functional expression of the mitochondrial pyruvate carrier. *Science* 337 93–96. 10.1126/science.1218530 22628554

[B120] Hipkiss A. R. (2019). Aging, Alzheimer’s disease and dysfunctional glycolysis; similar effects of too much and too little. *Aging Dis.* 10 1328–1331. 10.14336/AD.2019.0611 31788344 PMC6844594

[B121] Holscher C. (2018). Novel dual GLP-1/GIP receptor agonists show neuroprotective effects in Alzheimer’s and Parkinson’s disease models. *Neuropharmacology* 136 251–259. 10.1016/j.neuropharm.2018.01.040 29402504

[B122] Hoyer S. (2002). The aging brain. Changes in the neuronal insulin/insulin receptor signal transduction cascade trigger late-onset sporadic Alzheimer disease (SAD). A mini-review. *J. Neural Transm. (Vienna)* 109 991–1002. 10.1007/s007020200082 12111436

[B123] Hoyer S. Lee S. K. Loffler T. Schliebs R. (2000). Inhibition of the neuronal insulin receptor. An in vivo model for sporadic Alzheimer disease? *Ann. N. Y. Acad. Sci.* 920 256–258. 10.1111/j.1749-6632.2000.tb06932.x 11193160

[B124] Hyder F. Rothman D. L. Bennett M. R. (2013). Cortical energy demands of signaling and nonsignaling components in brain are conserved across mammalian species and activity levels. *Proc. Natl. Acad. Sci. U.S.A.* 110 3549–3554. 10.1073/pnas.1214912110 23319606 PMC3587194

[B125] Iadecola C. Nedergaard M. (2007). Glial regulation of the cerebral microvasculature. *Nat. Neurosci.* 10 1369–1376. 10.1038/nn2003 17965657

[B126] Imfeld P. Bodmer M. Jick S. S. Meier C. R. (2012). Metformin, other antidiabetic drugs, and risk of Alzheimer’s disease: a population-based case-control study. *J. Am. Geriatr. Soc.* 60 916–921. 10.1111/j.1532-5415.2012.03916.x 22458300

[B127] Jagust W. J. Landau S. M., and Alzheimer’s Disease Neuroimaging Initiative. (2012). Apolipoprotein E, not fibrillar beta-amyloid, reduces cerebral glucose metabolism in normal aging. *J. Neurosci.* 32 18227–18233. 10.1523/JNEUROSCI.3266-12.2012 23238736 PMC3537830

[B128] Jiang T. Cadenas E. (2014). Astrocytic metabolic and inflammatory changes as a function of age. *Aging Cell* 13 1059–1067. 10.1111/acel.12268 25233945 PMC4244278

[B129] Jiang T. Yin F. Yao J. Brinton R. D. Cadenas E. (2013). Lipoic acid restores age-associated impairment of brain energy metabolism through the modulation of Akt/JNK signaling and PGC1alpha transcriptional pathway. *Aging Cell* 12 1021–1031. 10.1111/acel.12127 23815272 PMC3819405

[B130] Jourdain P. Allaman I. Rothenfusser K. Fiumelli H. Marquet P. Magistretti P. J. (2016). L-Lactate protects neurons against excitotoxicity: implication of an ATP-mediated signaling cascade. *Sci. Rep.* 6:21250. 10.1038/srep21250 26893204 PMC4759786

[B131] Jourdain P. Rothenfusser K. Ben-Adiba C. Allaman I. Marquet P. Magistretti P. J. (2018). Dual action of L-Lactate on the activity of NR2B-containing NMDA receptors: from potentiation to neuroprotection. *Sci. Rep.* 8:13472. 10.1038/s41598-018-31534-y 30194439 PMC6128851

[B132] Karagiannis A. Gallopin T. Lacroix A. Plaisier F. Piquet J. Geoffroy H. (2021). Lactate is an energy substrate for rodent cortical neurons and enhances their firing activity. *Elife* 10:e71424. 10.7554/eLife.71424 34766906 PMC8651295

[B133] Kashiwaya Y. Bergman C. Lee J. H. Wan R. King M. T. Mughal M. R. (2013). A ketone ester diet exhibits anxiolytic and cognition-sparing properties, and lessens amyloid and tau pathologies in a mouse model of Alzheimer’s disease. *Neurobiol. Aging* 34 1530–1539. 10.1016/j.neurobiolaging.2012.11.023 23276384 PMC3619192

[B134] Keeney J. T. Ibrahimi S. Zhao L. (2015). Human ApoE isoforms differentially modulate glucose and amyloid metabolic pathways in female brain: evidence of the mechanism of neuroprotection by ApoE2 and implications for Alzheimer’s disease prevention and early intervention. *J. Alzheimers Dis.* 48 411–424. 10.3233/JAD-150348 26402005 PMC5485924

[B135] Kellar D. Craft S. (2020). Brain insulin resistance in Alzheimer’s disease and related disorders: mechanisms and therapeutic approaches. *Lancet Neurol.* 19 758–766. 10.1016/S1474-4422(20)30231-3 32730766 PMC9661919

[B136] Kelly T. Rose C. R. (2010). Ammonium influx pathways into astrocytes and neurones of hippocampal slices. *J. Neurochem.* 115 1123–1136. 10.1111/j.1471-4159.2010.07009.x 20854430

[B137] Koenig A. M. Mechanic-Hamilton D. Xie S. X. Combs M. F. Cappola A. R. Xie L. (2017). Effects of the Insulin Sensitizer metformin in Alzheimer disease: pilot data from a randomized placebo-controlled crossover study. *Alzheimer Dis. Assoc. Disord.* 31 107–113. 10.1097/WAD.0000000000000202 28538088 PMC5476214

[B138] Koss E. Friedland R. P. Ober B. A. Jagust W. J. (1985). Differences in lateral hemispheric asymmetries of glucose utilization between early- and late-onset Alzheimer-type dementia. *Am J. Psychiatry* 142 638–640. 10.1176/ajp.142.5.638 3872604

[B139] Krikorian R. Shidler M. D. Dangelo K. Couch S. C. Benoit S. C. Clegg D. J. (2012). Dietary ketosis enhances memory in mild cognitive impairment. *Neurobiol. Aging* 33 e419–e427. 10.1016/j.neurobiolaging.2010.10.006 21130529 PMC3116949

[B140] Krikorian R. Shidler M. D. Summer S. S. Sullivan P. G. Duker A. P. Isaacson R. S. (2019). Nutritional ketosis for mild cognitive impairment in Parkinson’s disease: a controlled pilot trial. *Clin. Park Relat. Disord.* 1 41–47. 10.1016/j.prdoa.2019.07.006 34316598 PMC8288565

[B141] Lauritzen K. H. Morland C. Puchades M. Holm-Hansen S. Hagelin E. M. Lauritzen F. (2014). Lactate receptor sites link neurotransmission, neurovascular coupling, and brain energy metabolism. *Cereb. Cortex* 24 2784–2795. 10.1093/cercor/bht136 23696276

[B142] Lee Y. Morrison B. M. Li Y. Lengacher S. Farah M. H. Hoffman P. N. (2012). Oligodendroglia metabolically support axons and contribute to neurodegeneration. *Nature* 487 443–448. 10.1038/nature11314 22801498 PMC3408792

[B143] Leino R. L. Gerhart D. Z. Duelli R. Enerson B. E. Drewes L. R. (2001). Diet-induced ketosis increases monocarboxylate transporter (MCT1) levels in rat brain. *Neurochem. Int.* 38 519–527. 10.1016/s0197-0186(00)00102-9 11248400

[B144] Lennox R. Porter D. W. Flatt P. R. Holscher C. Irwin N. Gault V. A. (2014). Comparison of the independent and combined effects of sub-chronic therapy with metformin and a stable GLP-1 receptor agonist on cognitive function, hippocampal synaptic plasticity and metabolic control in high-fat fed mice. *Neuropharmacology* 86 22–30. 10.1016/j.neuropharm.2014.06.026 24998752

[B145] Lerchundi R. Fernandez-Moncada I. Contreras-Baeza Y. Sotelo-Hitschfeld T. Machler P. Wyss M. T. (2015). NH4(+) triggers the release of astrocytic lactate via mitochondrial pyruvate shunting. *Proc. Natl. Acad. Sci. U.S.A.* 112 11090–11095. 10.1073/pnas.1508259112 26286989 PMC4568276

[B146] Lester-Coll N. Rivera E. J. Soscia S. J. Doiron K. Wands J. R. De La Monte S. M. (2006). Intracerebral streptozotocin model of type 3 diabetes: relevance to sporadic Alzheimer’s disease. *J. Alzheimers Dis.* 9 13–33. 10.3233/jad-2006-9102 16627931

[B147] Lev-Vachnish Y. Cadury S. Rotter-Maskowitz A. Feldman N. Roichman A. Illouz T. (2019). L-lactate promotes adult hippocampal neurogenesis. *Front Neurosci.* 13:403. 10.3389/fnins.2019.00403 PMC6542996 31178678

[B148] Li F. Sami A. Noristani H. N. Slattery K. Qiu J. Groves T. (2020). Glial metabolic rewiring promotes axon regeneration and functional recovery in the central nervous system. *Cell Metab.* 32 767–785.e7. 10.1016/j.cmet.2020.08.015 32941799 PMC7642184

[B149] Li Y. Duffy K. B. Ottinger M. A. Ray B. Bailey J. A. Holloway H. W. (2010). GLP-1 receptor stimulation reduces amyloid-beta peptide accumulation and cytotoxicity in cellular and animal models of Alzheimer’s disease. *J. Alzheimers Dis.* 19 1205–1219. 10.3233/JAD-2010-1314 20308787 PMC2948479

[B150] Li Y. Perry T. Kindy M. S. Harvey B. K. Tweedie D. Holloway H. W. (2009). GLP-1 receptor stimulation preserves primary cortical and dopaminergic neurons in cellular and rodent models of stroke and Parkinsonism. *Proc. Natl. Acad. Sci. U.S.A.* 106 1285–1290. 10.1073/pnas.0806720106 19164583 PMC2633544

[B151] Liang W. S. Reiman E. M. Valla J. Dunckley T. Beach T. G. Grover A. (2008). Alzheimer’s disease is associated with reduced expression of energy metabolism genes in posterior cingulate neurons. *Proc. Natl. Acad. Sci. U.S.A.* 105 4441–4446. 10.1073/pnas.0709259105 18332434 PMC2393743

[B152] Liddelow S. A. Guttenplan K. A. Clarke L. E. Bennett F. C. Bohlen C. J. Schirmer L. (2017). Neurotoxic reactive astrocytes are induced by activated microglia. *Nature* 541 481–487. 10.1038/nature21029 28099414 PMC5404890

[B153] Lin A. L. Jahrling J. B. Zhang W. Derosa N. Bakshi V. Romero P. (2017). Rapamycin rescues vascular, metabolic and learning deficits in apolipoprotein E4 transgenic mice with pre-symptomatic Alzheimer’s disease. *J. Cereb. Blood Flow Metab.* 37 217–226. 10.1177/0271678X15621575 26721390 PMC5167110

[B154] Liu L. Mackenzie K. R. Putluri N. Maletic-Savatic M. Bellen H. J. (2017). The glia-neuron lactate shuttle and elevated ROS promote lipid synthesis in neurons and lipid droplet accumulation in glia via APOE/D. *Cell Metab.* 26:e716. 10.1016/j.cmet.2017.08.024 28965825 PMC5677551

[B155] Liu W. Jalewa J. Sharma M. Li G. Li L. Holscher C. (2015). Neuroprotective effects of lixisenatide and liraglutide in the 1-methyl-4-phenyl-1,2,3,6-tetrahydropyridine mouse model of Parkinson’s disease. *Neuroscience* 303 42–50. 10.1016/j.neuroscience.2015.06.054 26141845

[B156] Lourenco M. V. Clarke J. R. Frozza R. L. Bomfim T. R. Forny-Germano L. Batista A. F. (2013). TNF-alpha mediates PKR-dependent memory impairment and brain IRS-1 inhibition induced by Alzheimer’s beta-amyloid oligomers in mice and monkeys. *Cell Metab.* 18 831–843. 10.1016/j.cmet.2013.11.002 24315369

[B157] Luchsinger J. A. Perez T. Chang H. Mehta P. Steffener J. Pradabhan G. (2016). Metformin in amnestic mild cognitive impairment: results of a pilot randomized placebo controlled clinical trial. *J. Alzheimers Dis.* 51 501–514. 10.3233/JAD-150493 26890736 PMC5079271

[B158] Machler P. Wyss M. T. Elsayed M. Stobart J. Gutierrez R. Von Faber-Castell A. (2016). In vivo evidence for a lactate gradient from astrocytes to neurons. *Cell Metab.* 23 94–102. 10.1016/j.cmet.2015.10.010 26698914

[B159] MacVicar B. A. Newman E. A. (2015). Astrocyte regulation of blood flow in the brain. *Cold Spring Harb. Perspect. Biol.* 7:a020388. 10.1101/cshperspect.a020388 25818565 PMC4448617

[B160] Magistretti P. Allaman I. (2016). *Bain Energy and Metabolism.* New York, NY: Springer.

[B161] Magistretti P. J. Allaman I. (2015). A cellular perspective on brain energy metabolism and functional imaging. *Neuron* 86 883–901. 10.1016/j.neuron.2015.03.035 25996133

[B162] Magistretti P. J. Allaman I. (2018). Lactate in the brain: from metabolic end-product to signalling molecule. *Nat. Rev. Neurosci.* 19 235–249. 10.1038/nrn.2018.19 29515192

[B163] Magistretti P. J. Chatton J. Y. (2005). Relationship between L-glutamate-regulated intracellular Na+ dynamics and ATP hydrolysis in astrocytes. *J. Neural Transm. (Vienna)* 112 77–85. 10.1007/s00702-004-0171-6 15599606

[B164] Magistretti P. J. Pellerin L. (1996). Cellular bases of brain energy metabolism and their relevance to functional brain imaging: evidence for a prominent role of astrocytes. *Cereb. Cortex* 6 50–61. 10.1093/cercor/6.1.50 8670638

[B165] Mahley R. W. Rall S. C. Jr. (2000). Apolipoprotein E: far more than a lipid transport protein. *Annu. Rev. Genomics Hum. Genet.* 1 507–537. 10.1146/annurev.genom.1.1.507 11701639

[B166] Mahley R. W. Weisgraber K. H. Huang Y. (2006). Apolipoprotein E4: a causative factor and therapeutic target in neuropathology, including Alzheimer’s disease. *Proc. Natl. Acad. Sci. U.S.A.* 103 5644–5651. 10.1073/pnas.0600549103 16567625 PMC1414631

[B167] Manninen T. Saudargiene A. Linne M. L. (2020). Astrocyte-mediated spike-timing-dependent long-term depression modulates synaptic properties in the developing cortex. *PLoS Comput. Biol.* 16:e1008360. 10.1371/journal.pcbi.1008360 PMC7654831 33170856

[B168] Marcus D. L. Freedman M. L. (1997). Decreased brain glucose metabolism in microvessels from patients with Alzheimer’s disease. *Ann. N. Y. Acad. Sci.* 826 248–253. 10.1111/j.1749-6632.1997.tb48476.x 9329696

[B169] Margineanu M. B. Mahmood H. Fiumelli H. Magistretti P. J. (2018). L-lactate regulates the expression of synaptic plasticity and neuroprotection genes in cortical neurons: a transcriptome analysis. *Front Mol Neurosci* 11:375. 10.3389/fnmol.2018.00375 PMC6191511 30364173

[B170] Masliah E. Mallory M. Hansen L. Deteresa R. Terry R. D. (1993). Quantitative synaptic alterations in the human neocortex during normal aging. *Neurology* 43 192–197. 10.1212/wnl.43.1_part_1.192 8423884

[B171] Mathiisen T. M. Lehre K. P. Danbolt N. C. Ottersen O. P. (2010). The perivascular astroglial sheath provides a complete covering of the brain microvessels: an electron microscopic 3D reconstruction. *Glia* 58 1094–1103. 10.1002/glia.20990 20468051

[B172] Mattay V. S. Fera F. Tessitore A. Hariri A. R. Berman K. F. Das S. (2006). Neurophysiological correlates of age-related changes in working memory capacity. *Neurosci. Lett.* 392 32–37. 10.1016/j.neulet.2005.09.025 16213083

[B173] McClean P. L. Jalewa J. Holscher C. (2015). Prophylactic liraglutide treatment prevents amyloid plaque deposition, chronic inflammation and memory impairment in APP/PS1 mice. *Behav. Brain Res.* 293 96–106. 10.1016/j.bbr.2015.07.024 26205827

[B174] McClean P. L. Parthsarathy V. Faivre E. Holscher C. (2011). The diabetes drug liraglutide prevents degenerative processes in a mouse model of Alzheimer’s disease. *J. Neurosci.* 31 6587–6594. 10.1523/JNEUROSCI.0529-11.2011 21525299 PMC6622662

[B175] McKenna M. C. (2007). The glutamate-glutamine cycle is not stoichiometric: fates of glutamate in brain. *J. Neurosci. Res.* 85 3347–3358. 10.1002/jnr.21444 17847118

[B176] McNeilly A. D. Williamson R. Balfour D. J. Stewart C. A. Sutherland C. (2012). A high-fat-diet-induced cognitive deficit in rats that is not prevented by improving insulin sensitivity with metformin. *Diabetologia* 55 3061–3070. 10.1007/s00125-012-2686-y 22898768

[B177] Merlini M. Meyer E. P. Ulmann-Schuler A. Nitsch R. M. (2011). Vascular beta-amyloid and early astrocyte alterations impair cerebrovascular function and cerebral metabolism in transgenic arcAbeta mice. *Acta Neuropathol.* 122 293–311. 10.1007/s00401-011-0834-y 21688176 PMC3168476

[B178] Minoshima S. Giordani B. Berent S. Frey K. A. Foster N. L. Kuhl D. E. (1997). Metabolic reduction in the posterior cingulate cortex in very early Alzheimer’s disease. *Ann. Neurol.* 42 85–94. 10.1002/ana.410420114 9225689

[B179] Mongeon R. Venkatachalam V. Yellen G. (2016). Cytosolic NADH-NAD(+) redox visualized in brain slices by two-photon fluorescence lifetime biosensor imaging. *Antioxid. Redox Signal.* 25 553–563. 10.1089/ars.2015.6593 26857245 PMC5041510

[B180] Mooradian A. D. Chung H. C. Shah G. N. (1997). GLUT-1 expression in the cerebra of patients with Alzheimer’s disease. *Neurobiol. Aging* 18 469–474. 10.1016/s0197-4580(97)00111-5 9390772

[B181] Moore R. Y. Bloom F. E. (1979). Central catecholamine neuron systems: anatomy and physiology of the norepinephrine and epinephrine systems. *Annu. Rev. Neurosci.* 2 113–168. 10.1146/annurev.ne.02.030179.000553 231924

[B182] Morland C. Lauritzen K. H. Puchades M. Holm-Hansen S. Andersson K. Gjedde A. (2015). The lactate receptor, G-protein-coupled receptor 81/hydroxycarboxylic acid receptor 1: expression and action in brain. *J. Neurosci. Res.* 93 1045–1055. 10.1002/jnr.23593 25881750

[B183] Muddapu V. R. Dharshini S. A. P. Chakravarthy V. S. Gromiha M. M. (2020). Neurodegenerative diseases - is metabolic deficiency the root cause? *Front. Neurosci.* 14:213. 10.3389/fnins.2020.00213 PMC7137637 32296300

[B184] Muraleedharan R. Gawali M. V. Tiwari D. Sukumaran A. Oatman N. Anderson J. (2020). AMPK-regulated astrocytic lactate shuttle plays a non-cell-autonomous role in neuronal survival. *Cell Rep.* 32:108092. 10.1016/j.celrep.2020.108092 32877674 PMC7531170

[B185] Nagele R. G. Wegiel J. Venkataraman V. Imaki H. Wang K. C. Wegiel J. (2004). Contribution of glial cells to the development of amyloid plaques in Alzheimer’s disease. *Neurobiol. Aging* 25 663–674. 10.1016/j.neurobiolaging.2004.01.007 15172746

[B186] Nehlig A. (2004). Brain uptake and metabolism of ketone bodies in animal models. *Prostaglandins Leukot. Essent. Fatty Acids* 70 265–275. 10.1016/j.plefa.2003.07.006 14769485

[B187] Newman L. A. Korol D. L. Gold P. E. (2011). Lactate produced by glycogenolysis in astrocytes regulates memory processing. *PLoS One* 6:e28427. 10.1371/journal.pone.0028427 PMC3236748 22180782

[B188] Novak P. Pimentel Maldonado D. A. Novak V. (2019). Safety and preliminary efficacy of intranasal insulin for cognitive impairment in Parkinson disease and multiple system atrophy: a double-blinded placebo-controlled pilot study. *PLoS One* 14:e0214364. 10.1371/journal.pone.0214364 PMC6483338 31022213

[B189] Oberheim N. A. Takano T. Han X. He W. Lin J. H. Wang F. (2009). Uniquely hominid features of adult human astrocytes. *J. Neurosci.* 29 3276–3287. 10.1523/JNEUROSCI.4707-08.2009 19279265 PMC2819812

[B190] Oksanen M. Petersen A. J. Naumenko N. Puttonen K. Lehtonen S. Gubert Olive M. (2017). PSEN1 mutant iPSC-derived model reveals severe astrocyte pathology in Alzheimer’s disease. *Stem Cell Rep.* 9 1885–1897. 10.1016/j.stemcr.2017.10.016 29153989 PMC5785689

[B191] Ong Q. R. Chan E. S. Lim M. L. Cole G. M. Wong B. S. (2014). Reduced phosphorylation of brain insulin receptor substrate and Akt proteins in apolipoprotein-E4 targeted replacement mice. *Sci. Rep.* 4:3754. 10.1038/srep03754 24435134 PMC3894554

[B192] Owen O. E. Morgan A. P. Kemp H. G. Sullivan J. M. Herrera M. G. Cahill G. F. Jr. (1967). Brain metabolism during fasting. *J. Clin. Invest.* 46 1589–1595. 10.1172/JCI105650 6061736 PMC292907

[B193] Palmer A. M. (1999). The activity of the pentose phosphate pathway is increased in response to oxidative stress in Alzheimer’s disease. *J. Neural Transm. (Vienna)* 106 317–328. 10.1007/s007020050161 10392540

[B194] Paoli A. Bianco A. Damiani E. Bosco G. (2014). Ketogenic diet in neuromuscular and neurodegenerative diseases. *Biomed. Res. Int.* 2014:474296. 10.1155/2014/474296 25101284 PMC4101992

[B195] Parikh H. Carlsson E. Chutkow W. A. Johansson L. E. Storgaard H. Poulsen P. (2007). TXNIP regulates peripheral glucose metabolism in humans. *PLoS Med.* 4:e158. 10.1371/journal.pmed.0040158 PMC1858708 17472435

[B196] Patching S. G. (2017). Glucose transporters at the blood-brain barrier: function, regulation and gateways for drug delivery. *Mol. Neurobiol.* 54 1046–1077. 10.1007/s12035-015-9672-6 26801191

[B197] Pawlosky R. J. Kemper M. F. Kashiwaya Y. King M. T. Mattson M. P. Veech R. L. (2017). Effects of a dietary ketone ester on hippocampal glycolytic and tricarboxylic acid cycle intermediates and amino acids in a 3xTgAD mouse model of Alzheimer’s disease. *J. Neurochem.* 141 195–207. 28099989 10.1111/jnc.13958 PMC5383517

[B198] Pellerin L. Magistretti P. J. (1994). Glutamate uptake into astrocytes stimulates aerobic glycolysis: a mechanism coupling neuronal activity to glucose utilization. *Proc. Natl. Acad. Sci. U.S.A.* 91 10625–10629. 10.1073/pnas.91.22.10625 7938003 PMC45074

[B199] Pellerin L. Magistretti P. J. (2012). Sweet sixteen for ANLS. *J. Cereb. Blood Flow Metab.* 32 1152–1166. 10.1038/jcbfm.2011.149 22027938 PMC3390819

[B200] Perry T. Greig N. H. (2005). Enhancing central nervous system endogenous GLP-1 receptor pathways for intervention in Alzheimer’s disease. *Curr. Alzheimer Res.* 2 377–385. 10.2174/1567205054367892 15974903

[B201] Perry T. Holloway H. W. Weerasuriya A. Mouton P. R. Duffy K. Mattison J. A. (2007). Evidence of GLP-1-mediated neuroprotection in an animal model of pyridoxine-induced peripheral sensory neuropathy. *Exp. Neurol.* 203 293–301. 10.1016/j.expneurol.2006.09.028 17125767 PMC1850958

[B202] Peterson A. C. Li C. R. (2018). Noradrenergic dysfunction in Alzheimer’s and Parkinson’s diseases-an overview of imaging studies. *Front. Aging Neurosci.* 10:127. 10.3389/fnagi.2018.00127 PMC5938376 29765316

[B203] Pintana H. Apaijai N. Pratchayasakul W. Chattipakorn N. Chattipakorn S. C. (2012). Effects of metformin on learning and memory behaviors and brain mitochondrial functions in high fat diet induced insulin resistant rats. *Life Sci.* 91 409–414. 10.1016/j.lfs.2012.08.017 22925597

[B204] Porras O. H. Ruminot I. Loaiza A. Barros L. F. (2008). Na(+)-Ca(2+) cosignaling in the stimulation of the glucose transporter GLUT1 in cultured astrocytes. *Glia* 56 59–68. 10.1002/glia.20589 17924581

[B205] Prapong T. Buss J. Hsu W. H. Heine P. West Greenlee H. Uemura E. (2002). Amyloid beta-peptide decreases neuronal glucose uptake despite causing increase in GLUT3 mRNA transcription and GLUT3 translocation to the plasma membrane. *Exp. Neurol.* 174 253–258. 10.1006/exnr.2001.7861 11922666

[B206] Puchalska P. Crawford P. A. (2017). Multi-dimensional roles of ketone bodies in fuel metabolism, signaling, and therapeutics. *Cell Metab.* 25 262–284. 10.1016/j.cmet.2016.12.022 28178565 PMC5313038

[B207] Qi L. Ke L. Liu X. Liao L. Ke S. Liu X. (2016). Subcutaneous administration of liraglutide ameliorates learning and memory impairment by modulating tau hyperphosphorylation via the glycogen synthase kinase-3beta pathway in an amyloid beta protein induced Alzheimer disease mouse model. *Eur. J. Pharmacol.* 783 23–32. 10.1016/j.ejphar.2016.04.052 27131827

[B208] Raber J. Huang Y. Ashford J. W. (2004). ApoE genotype accounts for the vast majority of AD risk and AD pathology. *Neurobiol. Aging* 25 641–650. 10.1016/j.neurobiolaging.2003.12.023 15172743

[B209] Rachmany L. Tweedie D. Li Y. Rubovitch V. Holloway H. W. Miller J. (2013). Exendin-4 induced glucagon-like peptide-1 receptor activation reverses behavioral impairments of mild traumatic brain injury in mice. *Age (Dordr)* 35 1621–1636. 10.1007/s11357-012-9464-0 22892942 PMC3776106

[B210] Raichle M. E. Mintun M. A. (2006). Brain work and brain imaging. *Annu. Rev. Neurosci.* 29 449–476. 10.1146/annurev.neuro.29.051605.112819 16776593

[B211] Rajkowska G. Stockmeier C. A. (2013). Astrocyte pathology in major depressive disorder: insights from human postmortem brain tissue. *Curr. Drug Targets* 14 1225–1236. 10.2174/13894501113149990156 23469922 PMC3799810

[B212] Rasmussen P. Wyss M. T. Lundby C. (2011). Cerebral glucose and lactate consumption during cerebral activation by physical activity in humans. *FASEB J.* 25 2865–2873. 10.1096/fj.11-183822 21602451

[B213] Reger M. A. Watson G. S. Green P. S. Baker L. D. Cholerton B. Fishel M. A. (2008a). Intranasal insulin administration dose-dependently modulates verbal memory and plasma amyloid-beta in memory-impaired older adults. *J. Alzheimers Dis.* 13 323–331. 10.3233/jad-2008-13309 18430999 PMC2804944

[B214] Reger M. A. Watson G. S. Green P. S. Wilkinson C. W. Baker L. D. Cholerton B. (2008b). Intranasal insulin improves cognition and modulates beta-amyloid in early AD. *Neurology* 70 440–448. 17942819 10.1212/01.WNL.0000265401.62434.36

[B215] Reiman E. M. Chen K. Alexander G. E. Caselli R. J. Bandy D. Osborne D. (2004). Functional brain abnormalities in young adults at genetic risk for late-onset Alzheimer’s dementia. *Proc. Natl. Acad. Sci. U.S.A.* 101 284–289. 10.1073/pnas.2635903100 14688411 PMC314177

[B216] Reiner D. J. Mietlicki-Baase E. G. Mcgrath L. E. Zimmer D. J. Bence K. K. Sousa G. L. (2016). Astrocytes regulate GLP-1 receptor-mediated effects on energy balance. *J. Neurosci.* 36 3531–3540. 27013681 10.1523/JNEUROSCI.3579-15.2016 PMC4804010

[B217] Resnick S. M. Pham D. L. Kraut M. A. Zonderman A. B. Davatzikos C. (2003). Longitudinal magnetic resonance imaging studies of older adults: a shrinking brain. *J. Neurosci.* 23 3295–3301. 10.1523/jneurosci.23-08-03295.2003 12716936 PMC6742337

[B218] Rivera E. J. Goldin A. Fulmer N. Tavares R. Wands J. R. De La Monte S. M. (2005). Insulin and insulin-like growth factor expression and function deteriorate with progression of Alzheimer’s disease: link to brain reductions in acetylcholine. *J. Alzheimers Dis.* 8 247–268. 16340083 10.3233/jad-2005-8304

[B219] Riveros N. Fiedler J. Lagos N. Munoz C. Orrego F. (1986). Glutamate in rat brain cortex synaptic vesicles: influence of the vesicle isolation procedure. *Brain Res.* 386 405–408. 2877717 10.1016/0006-8993(86)90181-2

[B220] Robinson M. M. Lowe V. J. Nair K. S. (2018). Increased brain glucose uptake after 12 weeks of aerobic high-intensity interval training in young and older adults. *J. Clin. Endocrinol. Metab.* 103 221–227. 10.1210/jc.2017-01571 29077855 PMC5761491

[B221] Rodriguez J. J. Olabarria M. Chvatal A. Verkhratsky A. (2009). Astroglia in dementia and Alzheimer’s disease. *Cell Death Differ* 16 378–385. 19057621 10.1038/cdd.2008.172

[B222] Roosterman D. Cottrell G. S. (2020). Astrocytes and neurons communicate via a monocarboxylic acid shuttle. *AIMS Neurosci.* 7 94–106. 10.3934/Neuroscience.2020007 32607414 PMC7321766

[B223] Ros J. Pecinska N. Alessandri B. Landolt H. Fillenz M. (2001). Lactate reduces glutamate-induced neurotoxicity in rat cortex. *J. Neurosci. Res.* 66 790–794. 10.1002/jnr.10043 11746403

[B224] Rosenbloom M. Barclay T. R. Kashyap B. Hage L. O’keefe L. R. Svitak A. (2021). A phase ii, single-center, randomized, double-blind, placebo-controlled study of the safety and therapeutic efficacy of intranasal glulisine in amnestic mild cognitive impairment and probable mild Alzheimer’s disease. *Drugs Aging* 38 407–415. 10.1007/s40266-021-00845-7 33719017

[B225] Rotermund C. Machetanz G. Fitzgerald J. C. (2018). The therapeutic potential of metformin in neurodegenerative diseases. *Front. Endocrinol. (Lausanne)* 9:400. 10.3389/fendo.2018.00400 PMC6060268 30072954

[B226] Roumes H. Jolle C. Blanc J. Benkhaled I. Chatain C. P. Massot P. (2021). Lactate transporters in the rat barrel cortex sustain whisker-dependent BOLD fMRI signal and behavioral performance. *Proc. Natl. Acad. Sci. U.S.A.* 118:e2112466118. 10.1073/pnas.2112466118 34782470 PMC8617497

[B227] Rub U. Del Tredici K. Schultz C. Thal D. R. Braak E. Braak H. (2001). The autonomic higher order processing nuclei of the lower brain stem are among the early targets of the Alzheimer’s disease-related cytoskeletal pathology. *Acta Neuropathol.* 101 555–564. 10.1007/s004010000320 11515783

[B228] Saab A. S. Tzvetavona I. D. Trevisiol A. Baltan S. Dibaj P. Kusch K. (2016). Oligodendroglial NMDA receptors regulate glucose import and axonal energy metabolism. *Neuron* 91 119–132. 10.1016/j.neuron.2016.05.016 27292539 PMC9084537

[B229] Sada N. Lee S. Katsu T. Otsuki T. Inoue T. (2015). Epilepsy treatment. Targeting LDH enzymes with a stiripentol analog to treat epilepsy. *Science* 347 1362–1367. 10.1126/science.aaa1299 25792327

[B230] Salat D. H. Buckner R. L. Snyder A. Z. Greve D. N. Desikan R. S. Busa E. (2004). Thinning of the cerebral cortex in aging. *Cereb. Cortex* 14 721–730. 15054051 10.1093/cercor/bhh032

[B231] Samaras K. Makkar S. Crawford J. D. Kochan N. A. Wen W. Draper B. (2020). Metformin use is associated with slowed cognitive decline and reduced incident dementia in older adults with type 2 diabetes: the sydney memory and ageing study. *Diabetes Care* 43 2691–2701. 10.2337/dc20-0892 32967921

[B232] Schurr A. Payne R. S. Miller J. J. Rigor B. M. (1997). Glia are the main source of lactate utilized by neurons for recovery of function posthypoxia. *Brain Res.* 774 221–224. 10.1016/s0006-8993(97)81708-8 9452213

[B233] Schurr A. Payne R. S. Miller J. J. Tseng M. T. Rigor B. M. (2001). Blockade of lactate transport exacerbates delayed neuronal damage in a rat model of cerebral ischemia. *Brain Res.* 895 268–272. 11259789 10.1016/s0006-8993(01)02082-0

[B234] Simpson I. A. Chundu K. R. Davies-Hill T. Honer W. G. Davies P. (1994). Decreased concentrations of GLUT1 and GLUT3 glucose transporters in the brains of patients with Alzheimer’s disease. *Ann. Neurol.* 35 546–551. 8179300 10.1002/ana.410350507

[B235] Sluggett J. K. Koponen M. Bell J. S. Taipale H. Tanskanen A. Tiihonen J. (2020). Metformin and risk of Alzheimer’s disease among community-dwelling people with diabetes: a national case-control study. *J. Clin. Endocrinol. Metab.* 105:dgz234. 10.1210/clinem/dgz234 31778170

[B236] Smith D. Pernet A. Hallett W. A. Bingham E. Marsden P. K. Amiel S. A. (2003). Lactate: a preferred fuel for human brain metabolism in vivo. *J. Cereb. Blood Flow Metab.* 23 658–664. 10.1097/01.WCB.0000063991.19746.11 12796713

[B237] Sowell E. R. Peterson B. S. Thompson P. M. Welcome S. E. Henkenius A. L. Toga A. W. (2003). Mapping cortical change across the human life span. *Nat. Neurosci.* 6 309–315. 10.1038/nn1008 12548289

[B238] Steen E. Terry B. M. Rivera E. J. Cannon J. L. Neely T. R. Tavares R. (2005). Impaired insulin and insulin-like growth factor expression and signaling mechanisms in Alzheimer’s disease–is this type 3 diabetes? *J. Alzheimers Dis.* 7 63–80. 10.3233/jad-2005-7107 15750215

[B239] Sullivan P. G. Rippy N. A. Dorenbos K. Concepcion R. C. Agarwal A. K. Rho J. M. (2004). The ketogenic diet increases mitochondrial uncoupling protein levels and activity. *Ann. Neurol.* 55 576–580. 10.1002/ana.20062 15048898

[B240] Supplie L. M. Duking T. Campbell G. Diaz F. Moraes C. T. Gotz M. (2017). Respiration-deficient astrocytes survive as glycolytic cells in vivo. *J. Neurosci.* 37 4231–4242. 10.1523/JNEUROSCI.0756-16.2017 28314814 PMC6596567

[B241] Suzuki A. Stern S. A. Bozdagi O. Huntley G. W. Walker R. H. Magistretti P. J. (2011). Astrocyte-neuron lactate transport is required for long-term memory formation. *Cell* 144 810–823. 10.1016/j.cell.2011.02.018 21376239 PMC3073831

[B242] Talbot K. Wang H. Y. Kazi H. Han L. Y. Bakshi K. P. Stucky A. (2012). Demonstrated brain insulin resistance in Alzheimer’s disease patients is associated with IGF-1 resistance, IRS-1 dysregulation, and cognitive decline. *J. Clin. Invest.* 122 1316–1338. 10.1172/JCI59903 22476197 PMC3314463

[B243] Tang B. L. (2020). Glucose, glycolysis, and neurodegenerative diseases. *J. Cell. Physiol.* 235 7653–7662. 10.1002/jcp.29682 32239718

[B244] Taouis M. Torres-Aleman I. (2019). Editorial: Insulin and The Brain. *Front Endocrinol (Lausanne)* 10:299. 10.3389/fendo.2019.00299 PMC6524151 31133989

[B245] ThomasS. C.AlhasawiA.AppannaV. P.AugerC.AppannaV. D. (2015). Brain metabolism and Alzheimer’s disease: the prospect of a metabolite-based therapy. *J. Nutr. Health Aging* 19 58–63. 10.1007/s12603-014-0511-7 25560817

[B246] Tieu K. Perier C. Caspersen C. Teismann P. Wu D. C. Yan S. D. (2003). D-beta-hydroxybutyrate rescues mitochondrial respiration and mitigates features of Parkinson disease. *J. Clin. Invest.* 112 892–901. 10.1172/JCI18797 12975474 PMC193668

[B247] Tomi M. Zhao Y. Thamotharan S. Shin B. C. Devaskar S. U. (2013). Early life nutrient restriction impairs blood-brain metabolic profile and neurobehavior predisposing to Alzheimer’s disease with aging. *Brain Res.* 1495 61–75. 10.1016/j.brainres.2012.11.050 23228723 PMC4174601

[B248] Uemura E. Greenlee H. W. (2001). Amyloid beta-peptide inhibits neuronal glucose uptake by preventing exocytosis. *Exp. Neurol.* 170 270–276. 10.1006/exnr.2001.7719 11476592

[B249] Vadini F. Simeone P. G. Boccatonda A. Guagnano M. T. Liani R. Tripaldi R. (2020). Liraglutide improves memory in obese patients with prediabetes or early type 2 diabetes: a randomized, controlled study. *Int. J. Obes. (Lond.).* 44 1254–1263. 10.1038/s41366-020-0535-5 31965072

[B250] Van der Auwera I. Wera S. Van Leuven F. Henderson S. T. (2005). A ketogenic diet reduces amyloid beta 40 and 42 in a mouse model of Alzheimer’s disease. *Nutr. Metab. (Lond.).* 2:28. 10.1186/1743-7075-2-28 16229744 PMC1282589

[B251] van Gijsel-Bonnello M. Baranger K. Benech P. Rivera S. Khrestchatisky M. De Reggi M. (2017). Metabolic changes and inflammation in cultured astrocytes from the 5xFAD mouse model of Alzheimer’s disease: alleviation by pantethine. *PLoS One* 12:e0175369. 10.1371/journal.pone.0175369 PMC5391924 28410378

[B252] Vandoorne T. De Bock K. Van Den Bosch L. (2018). Energy metabolism in ALS: an underappreciated opportunity? *Acta Neuropathol.* 135 489–509. 10.1007/s00401-018-1835-x 29549424 PMC5978930

[B253] Vardjan N. Chowdhury H. H. Horvat A. Velebit J. Malnar M. Muhic M. (2018). Enhancement of astroglial aerobic glycolysis by extracellular lactate-mediated increase in cAMP. *Front. Mol. Neurosci.* 11:148. 10.3389/fnmol.2018.00148 PMC5953330 29867342

[B254] Veech R. L. (2004). The therapeutic implications of ketone bodies: the effects of ketone bodies in pathological conditions: ketosis, ketogenic diet, redox states, insulin resistance, and mitochondrial metabolism. *Prostaglandins Leukot. Essent. Fatty Acids* 70 309–319. 10.1016/j.plefa.2003.09.007 14769489

[B255] Wang J. Gallagher D. Devito L. M. Cancino G. I. Tsui D. He L. (2012). Metformin activates an atypical PKC-CBP pathway to promote neurogenesis and enhance spatial memory formation. *Cell Stem Cell* 11 23–35. 10.1016/j.stem.2012.03.016 22770240

[B256] Watson K. T. Wroolie T. E. Tong G. Foland-Ross L. C. Frangou S. Singh M. (2019). Neural correlates of liraglutide effects in persons at risk for Alzheimer’s disease. *Behav. Brain Res.* 356 271–278. 30099030 10.1016/j.bbr.2018.08.006

[B257] Weise C. M. Chen K. Chen Y. Kuang X. Savage C. R. Reiman E. M. (2018). Left lateralized cerebral glucose metabolism declines in amyloid-beta positive persons with mild cognitive impairment. *Neuroimage Clin.* 20 286–296. 10.1016/j.nicl.2018.07.016 30101060 PMC6084012

[B258] Westhaus A. Blumrich E. M. Dringen R. (2017). The antidiabetic drug metformin stimulates glycolytic lactate production in cultured primary rat astrocytes. *Neurochem. Res.* 42 294–305. 10.1007/s11064-015-1733-8 26433380

[B259] Williams H. C. Farmer B. C. Piron M. A. Walsh A. E. Bruntz R. C. Gentry M. S. (2020). APOE alters glucose flux through central carbon pathways in astrocytes. *Neurobiol. Dis.* 136:104742. 10.1016/j.nbd.2020.104742 31931141 PMC7044721

[B260] Wilson R. S. Nag S. Boyle P. A. Hizel L. P. Yu L. Buchman A. S. (2013). Neural reserve, neuronal density in the locus ceruleus, and cognitive decline. *Neurology* 80 1202–1208. 10.1212/WNL.0b013e3182897103 23486878 PMC3691778

[B261] Wong-Riley M. T. (1989). Cytochrome oxidase: an endogenous metabolic marker for neuronal activity. *Trends Neurosci.* 12 94–101. 2469224 10.1016/0166-2236(89)90165-3

[B262] Woo B. K. Harwood D. G. Melrose R. J. Mandelkern M. A. Campa O. M. Walston A. (2010). Executive deficits and regional brain metabolism in Alzheimer’s disease. *Int. J. Geriatr. Psychiatry* 25 1150–1158. 20069587 10.1002/gps.2452

[B263] Wu L. Zhang X. Zhao L. (2018). Human ApoE Isoforms differentially modulate brain glucose and ketone body metabolism: implications for Alzheimer’s disease risk reduction and early intervention. *J. Neurosci.* 38 6665–6681. 10.1523/JNEUROSCI.2262-17.2018 29967007 PMC6067075

[B264] Xu P. T. Schmechel D. Rothrock-Christian T. Burkhart D. S. Qiu H. L. Popko B. (1996). Human apolipoprotein E2, E3, and E4 isoform-specific transgenic mice: human-like pattern of glial and neuronal immunoreactivity in central nervous system not observed in wild-type mice. *Neurobiol. Dis.* 3 229–245. 10.1006/nbdi.1996.0023 8980023

[B265] Xu Q. Bernardo A. Walker D. Kanegawa T. Mahley R. W. Huang Y. (2006). Profile and regulation of apolipoprotein E (ApoE) expression in the CNS in mice with targeting of green fluorescent protein gene to the ApoE locus. *J. Neurosci.* 26 4985–4994. 10.1523/JNEUROSCI.5476-05.2006 16687490 PMC6674234

[B266] Xu Q. Zhang Y. Zhang X. Liu L. Zhou B. Mo R. (2020). Medium-chain triglycerides improved cognition and lipid metabolomics in mild to moderate Alzheimer’s disease patients with APOE4(-/-): a double-blind, randomized, placebo-controlled crossover trial. *Clin. Nutr.* 39 2092–2105. 10.1016/j.clnu.2019.10.017 31694759

[B267] Yan X. Hu Y. Wang B. Wang S. Zhang X. (2020). Metabolic dysregulation contributes to the progression of Alzheimer’s disease. *Front. Neurosci.* 14:530219. 10.3389/fnins.2020.530219 PMC7674854 33250703

[B268] Yang J. Ruchti E. Petit J. M. Jourdain P. Grenningloh G. Allaman I. (2014). Lactate promotes plasticity gene expression by potentiating NMDA signaling in neurons. *Proc. Natl. Acad. Sci. U.S.A.* 111 12228–12233. 10.1073/pnas.1322912111 25071212 PMC4143009

[B269] Yang Y. Bender A. R. Raz N. (2015). Age related differences in reaction time components and diffusion properties of normal-appearing white matter in healthy adults. *Neuropsychologia* 66 246–258. 10.1016/j.neuropsychologia.2014.11.020 25460349 PMC4306435

[B270] Yao J. Brinton R. D. (2011). Targeting mitochondrial bioenergetics for Alzheimer’s prevention and treatment. *Curr. Pharm. Des.* 17 3474–3479. 10.2174/138161211798072517 21902662 PMC4209948

[B271] Yildirim Simsir I. Soyaltin U. E. Cetinkalp S. (2018). Glucagon like peptide-1 (GLP-1) likes Alzheimer’s disease. *Diabetes Metab. Syndr.* 12 469–475. 10.1016/j.dsx.2018.03.002 29598932

[B272] Yin F. Sancheti H. Patil I. Cadenas E. (2016). Energy metabolism and inflammation in brain aging and Alzheimer’s disease. *Free Radic. Biol. Med.* 100 108–122. 10.1016/j.freeradbiomed.2016.04.200 27154981 PMC5094909

[B273] Yu Y. Herman P. Rothman D. L. Agarwal D. Hyder F. (2018). Evaluating the gray and white matter energy budgets of human brain function. *J. Cereb. Blood Flow Metab.* 38 1339–1353. 10.1177/0271678X17708691 28589753 PMC6092772

[B274] Zhang J. Cao Q. Li S. Lu X. Zhao Y. Guan J. S. (2013). 3-Hydroxybutyrate methyl ester as a potential drug against Alzheimer’s disease via mitochondria protection mechanism. *Biomaterials* 34 7552–7562. 10.1016/j.biomaterials.2013.06.043 23849878

[B275] Zhao N. Liu C. C. Van Ingelgom A. J. Martens Y. A. Linares C. Knight J. A. (2017). Apolipoprotein E4 impairs neuronal insulin signaling by trapping insulin receptor in the endosomes. *Neuron* 96 115–129.e5. 10.1016/j.neuron.2017.09.003 28957663 PMC5621659

[B276] Zhao W. Varghese M. Vempati P. Dzhun A. Cheng A. Wang J. (2012). Caprylic triglyceride as a novel therapeutic approach to effectively improve the performance and attenuate the symptoms due to the motor neuron loss in ALS disease. *PLoS One* 7:e49191. 10.1371/journal.pone.0049191 PMC3492315 23145119

[B277] Zhao W. Q. Townsend M. (2009). Insulin resistance and amyloidogenesis as common molecular foundation for type 2 diabetes and Alzheimer’s disease. *Biochim. Biophys. Acta* 1792 482–496. 10.1016/j.bbadis.2008.10.014 19026743

[B278] Zheng J. Xie Y. Ren L. Qi L. Wu L. Pan X. (2021). GLP-1 improves the supportive ability of astrocytes to neurons by promoting aerobic glycolysis in Alzheimer’s disease. *Mol. Metab.* 47:101180. 10.1016/j.molmet.2021.101180 33556642 PMC7905479

[B279] Zhou J. Liu T. Guo H. Cui H. Li P. Feng D. (2018). Lactate potentiates angiogenesis and neurogenesis in experimental intracerebral hemorrhage. *Exp. Mol. Med.* 50 1–12. 10.1038/s12276-018-0113-2 29980670 PMC6035243

[B280] Zulfiqar S. Garg P. Nieweg K. (2019). Contribution of astrocytes to metabolic dysfunction in the Alzheimer’s disease brain. *Biol. Chem.* 400 1113–1127. 10.1515/hsz-2019-0140 31188740

