# Erratum: Astrocytes as Key Regulators of Brain Energy Metabolism: New Therapeutic Perspectives

**DOI:** 10.3389/fphys.2022.867827

**Published:** 2022-02-25

**Authors:** Frontiers Production Office

**Affiliations:** Frontiers Media SA, Lausanne, Switzerland

**Keywords:** astrocytes, lactate, glucose, brain, energy, metabolism, new therapeutic approach, GliaPharm

Due to a production error, PM was listed as an employee for GliaPharm SA in the Conflict of Interest statement. PM is an advisor for GliaPharm SA. The publisher apologizes for this mistake.

The original article has been updated.

